# CardioMEA: comprehensive data analysis platform for studying cardiac diseases and drug responses

**DOI:** 10.3389/fphys.2024.1472126

**Published:** 2024-10-30

**Authors:** Jihyun Lee, Eliane Duperrex, Ibrahim El-Battrawy, Alyssa Hohn, Ardan M. Saguner, Firat Duru, Vishalini Emmenegger, Lukas Cyganek, Andreas Hierlemann, Hasan Ulusan

**Affiliations:** ^1^ Department of Biosystems Science and Engineering, ETH Zürich, Basel, Switzerland; ^2^ St. Josef Hospital, Ruhr-University Bochum, Bochum, Germany; ^3^ German Center for Cardiovascular Research, Partner Site Heidelberg-Mannheim and Göttingen, Heidelberg, Germany; ^4^ Department of Molecular and Experimental Cardiology, Institut für Forschung und Lehre (IFL), Ruhr-University Bochum, Bochum, Germany; ^5^ Institute of Physiology, Ruhr-University Bochum, Bochum, Germany; ^6^ Department of Cardiology, Electrophysiology Division, University Heart Center Zurich, Zurich, Switzerland; ^7^ Center for Translational and Experimental Cardiology (CTEC), University of Zürich, Zurich, Switzerland; ^8^ Stem Cell Unit, Clinic for Cardiology and Pneumology, University Medical Center Göttingen, Göttingen, Germany

**Keywords:** cardiac arrhythmia, microelectrode array, machine learning, antiarrhythmic drug, induced pluripotent stem cell

## Abstract

**Introduction:**

In recent years, high-density microelectrode arrays (HD-MEAs) have emerged as a valuable tool in preclinical research for characterizing the electrophysiology of human induced pluripotent stem-cell-derived cardiomyocytes (iPSC-CMs). HD-MEAs enable the capturing of both extracellular and intracellular signals on a large scale, while minimizing potential damage to the cell. However, despite technological advancements of HD-MEAs, there is a lack of effective data-analysis platforms that are capable of processing and analyzing the data, particularly in the context of cardiac arrhythmias and drug testing.

**Methods:**

To address this need, we introduce CardioMEA, a comprehensive data-analysis platform designed specifically for HD-MEA data that have been obtained from iPSCCMs. CardioMEA features scalable data processing pipelines and an interactive web-based dashboard for advanced visualization and analysis. In addition to its core functionalities, CardioMEA incorporates modules designed to discern crucial electrophysiological features between diseased and healthy iPSC-CMs. Notably, CardioMEA has the unique capability to analyze both extracellular and intracellular signals, thereby facilitating customized analyses for specific research tasks.

**Results and discussion:**

We demonstrate the practical application of CardioMEA by analyzing electrophysiological signals from iPSC-CM cultures exposed to seven antiarrhythmic drugs. CardioMEA holds great potential as an intuitive, userfriendly platform for studying cardiac diseases and assessing drug effects.

## Introduction

As the principal cause of mortality in the Western world, cardiovascular diseases represent a significant healthcare challenge (Multiple Cause, 2024). Moreover, medication intended for non-cardiac conditions can inadvertently induce life-threatening arrhythmias due to off-target effects on the heart (Pai and Nahata, 2000; Singal and Iliskovic, 1998). Therefore, preclinical research to investigate drug effects on the heart is of paramount importance. Preclinical testing helps to evaluate the therapeutic potential and to identify any harmful effects of drug candidates targeted at cardiac diseases before commencing human trials.

A combination of human induced pluripotent stem cell (iPSC) and microelectrode array (MEA) technologies has been effectively employed to characterize disease phenotypes and evaluate drug responses *in vitro*. Measurements of either extracellular field potentials (Yamamoto et al., 2016; Blinova et al., 2018; Li et al., 2020; Mulder et al., 2018) or intracellular-like signals (Hayes et al., 2019; Iachetta et al., 2023; Lee et al., 2022) emanating from iPSC-derived cardiomyocytes (CMs) have been conducted. High-density MEAs (HD-MEAs) offer several advantages over conventional MEAs featuring larger gold or TiO2 electrodes (>40 µm diameter) at a considerably larger pitch (>250 µm). First, the co-integration with signal-conditioning circuitry provides better signal-to-noise characteristics (signal-to-noise ratio (SNR)) (Ballini et al., 2014), as the signals are filtered, amplified, and digitized right at the site of biological signal generation. Featuring a large number of small electrodes at high spatial density, HD-MEAs can more accurately capture local characteristics of field potentials, which enables a more precise detection of cardiac signal signatures and propagation characteristics. The smaller, more densely packed electrodes help to effectively capture locally more confined extracellular field potential signals and to reduce interference from cell movement artifacts. Additionally, a larger number of measurements and measurement values becomes available to reliably characterize a certain cell preparation. The co-integration of electrodes and circuity helps to reduce noise interference along the leads and connections and to minimize environmental disturbances due to the fully differential amplifier architectures and the fact that all amplifiers sit on the same substrate with the cells. The fully programmable filter and amplifier cascade including digitalization on the same HD-MEA chip helps to minimize artifacts and enables effective common-mode rejection. The high spatial resolution, enabled by the dense arrangement of the electrodes (Emmenegger et al., 2019; Müller et al., 2015), allows for a precise mapping of field potential propagation across cardiomyocyte cultures, which is critical for studying wavefronts, conduction velocity, and arrhythmic events.

Over the past decade, advancements in micro- and nanotechnology have pushed the boundaries of HD-MEA technology, with an ever increasing electrode density and number of readout channels generating large data volumes. Despite significant progress in HD-MEA technology, platforms to process and interpret this data remain scarce. Previous studies have offered MATLAB-based software using graphical user interfaces (GUIs) for processing and visualizing MEA data (Pradhapan et al., 2013; Georgiadis et al., 2015). While GUI applications provide a user-friendly interface and direct interaction with the operating system for efficient computation, they have limitations in the adaptability of the data-processing steps. If users wish to alter processing steps or handle varying file formats, proficiency in GUI programming becomes an essential prerequisite. Additionally, MATLAB is a commercial software, which generates additional costs, when code alterations are necessary. Cardio PyMEA, an open-source application, was recently proposed to address some of these issues (Dunham et al., 2022). Built on Python, a widely accessible and extensively used programming language, Cardio PyMEA offers the potential to serve a broad user base. However, the authors of Cardio PyMEA reported performance issues, including the GUI freezing during data processing due to Python’s Global Interpreter Lock (GIL). They also noted data processing speed as a limitation.

While there has been considerable progress in the development of analysis platforms for MEA data of CMs, there is still a lot of work to be done to achieve comprehensive characterization of the electrophysiological characteristics of healthy and diseased cells or potential drug responses. Most existing platforms have been designed for working with MEAs that feature a comparably low number of electrodes and offer low spatial resolution, which poses a challenge given the fact that state-of-the-art HD-MEAs feature thousands of readout electrodes and channels (Ballini et al., 2014; Yuan et al., 2020; Dragas et al., 2017) and have an electrode pitch as small as 11.47 µm (Suzuki et al., 2023). HD-MEAs generate massive numbers of data points per experiment, so that a statistically relevant sample size can be quickly reached. Moreover, due to the high density and large number of electrodes, they enable reliable signal-conduction-speed estimation of the CM cell assembly (Bayly et al., 1998).

Commercially available MEA systems typically include proprietary software tools that offer basic data processing and visualization capabilities. CardioMEA offers several advantages over commercial software tools, primarily due to its open-source nature, which allows for free adaptation and expansion to accommodate and support advanced data analysis methods. Commercial software often lacks transparency regarding the algorithms used for analysis and is typically designed for more generic applications, catering to a broader customer base rather than to specific research needs. Moreover, the use of commercial software is usually limited to data acquired by the respective proprietary MEA systems. In contrast, open-source platforms like CardioMEA are highly adaptable, offering great flexibility for adding new features or new data formats whenever needed.

Most of the currently used data-analysis platforms have been designed for processing and analysis of extracellular signals and do not fully leverage the capabilities of HD-MEAs, as recent studies have shown that HD-MEAs are capable of recording not only extracellular but also intracellular-like signals on demand (Lee et al., 2022; Abbott et al., 2017; Iachetta et al., 2021). Compared to intracellular-like measurements, the measurement of merely extracellular activities may not be sufficient to adequately capture drug-induced alterations in cardiac membrane potentials (Spira and Hai, 2013). In most existing platforms, data processing and visualization steps are integrated into a single pipeline. However, this integration leads to inefficiencies in comparative analysis across multiple data files, as each file requires a significant amount of time to process.

For a comprehensive evaluation, an analysis platform should enable the comparison of drug responses at different concentrations or across multiple cell lines with adequate visualization. Furthermore, data processing should be efficient and make optimal use of available computational resources, such as multiple central processing units (CPUs) and memory space, which are often not needed for visualization and comparative analysis. Therefore, the execution of all analysis steps in a single pipeline does not provide optimal performance.

**FIGURE 1:**
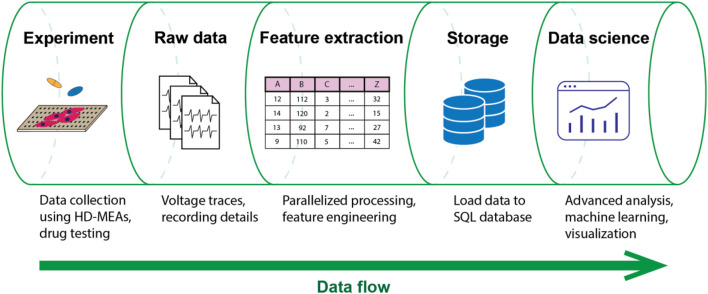
Data flow in the CardioMEA data analysis platform. CardioMEA incorporates every data analysis stage within its structure, ranging from an initial identification of experimental recording files, through raw-data handling and feature extraction, to the subsequent data uploading to an SQL database. Further, it provides a robust system for data visualization and advanced analysis, offering a comprehensive solution for evaluating and interpreting experimental data.

To overcome the aforementioned challenges and limitations, we developed CardioMEA, a comprehensive data analysis platform providing a set of pipelines for the extraction of raw HD-MEA data, feature extraction, data storage, visualization, and advanced analysis (Figure 1). CardioMEA includes data-processing pipelines and a web-based dashboard for data visualization and feature analysis, which have been designed to ensure reproducibility, scalability, and maintainability of all processing tasks. Numerous data files can be processed in parallel using multiple CPUs, which entails a significant reduction in computation time. The resulting processed data are stored in a structured query language (SQL) database, enabling tracking of data history and previous processing steps. In addition, querying pre-processed data from the database facilitates comparative analysis by eliminating the need for repetitive and time-consuming processing steps during each access. Moreover, the CardioMEA Dashboard offers an interactive, web-based platform for data visualization and analysis. This “no-code” application is designed to benefit a broad range of users, facilitating exploratory data analysis, while only a minimal effort is required to understand the underlying code. We demonstrate that - with just a few mouse clicks within the CardioMEA Dashboard - it is possible to analyze CM data of three iPSC lines and their responses to seven antiarrhythmic drugs.

CardioMEA offers unique functionalities for feature analysis, enabling users to assess the predictive power and contribution of each feature in classifying data collected from healthy and diseased cells, as demonstrated in this study. In addition, CardioMEA presents the first open-source platform that can also process and visualize intracellular-like signals of CMs, recorded with HD-MEAs, to obtain detailed insights into membrane potential dynamics (Spira and Hai, 2013). Its open-source configuration and standardized structure may empower a broad spectrum of users in cardiology and the pharmaceutical sector to effortlessly implement and adjust the platform according to their specific requirements. CardioMEA has been designed for use by scientists with little or no experience in programming, supporting their efforts to investigate the efficacy or potential toxicity of compounds.

As the field of cardiac disease, toxicity research, and individualized precision medicine continues to expand - with ever-increasing data volumes - the need for efficient, scalable, and user-friendly data-analysis tools will grow correspondingly. CardioMEA holds significant potential for drug development and personalized medicine, offering to assess drug effects and characterize patient-derived CMs based on intracellular and extracellular signals. By efficiently streamlining intrinsic processes and featuring an interactive dashboard for data visualization and advanced analysis, CardioMEA will help to advance biomedical research and the development of therapeutics.

## Materials and methods

### Data science framework to build the data pipeline

For the development of an open-source data analysis platform, adherence to software engineering best practices is essential to ensure that the code is both readily comprehensible and maintainable. Furthermore, the possibility of reproducing data processing and analysis is a crucial consideration in constructing such a platform. To meet these requirements, we utilized Kedro (Alam, 2024), a Python-based open-source framework renowned for fostering the development of modular and maintainable data science platforms. Kedro comes with built-in wrappers that manage input and output data in diverse formats, including comma-separated values (CSV) and SQL. These features enable efficient data extraction, transformation, and loading processes - critical aspects that make Kedro a suitable tool for developing the data pipelines for this study. By constructing pipelines, which contain sequentially chained nodes, we created a data-flow structure that is both easy to comprehend and reproducible. Kedro’s pipeline-based architecture enabled us to break down the data processing tasks into smaller, reusable components (nodes). The modularity simplifies the addition of new features or steps without disrupting the entire workflow, which is particularly advantageous for researchers wishing to expand existing analyses, to incorporate additional data sources, or to explore new hypotheses within the same project framework. The principles of clarity and reproducibility were consistently prioritized in our platform’s design and functionality.

### Feature extraction algorithms

**FIGURE 2:**
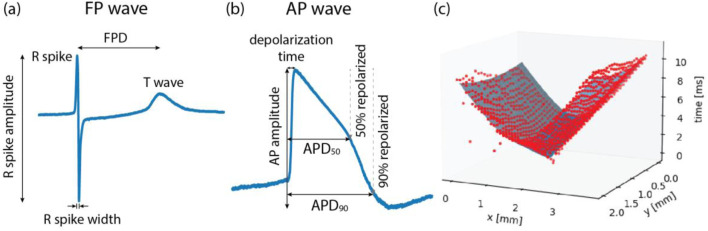
Illustrations of **(A)** field potential (FP) wave features, **(B)** action potential (AP) wave features, and **(C)** conduction speed estimation. FP waves are obtained from extracellular measurements and AP waves from intracellular or intracellular-like measurements. Field potential duration (FPD) is the time difference between the R spike and the T wave. Action potential duration (APD) is the time taken from the onset of depolarization until 50% repolarization (APD50) or 90% repolarization (APD90). **(C)** To estimate the conduction speed, data points (red color), collected from FP recordings, were fitted to a cone-shaped surface (blue color). Each red dot represents FP data from one electrode.

To extract features from the raw data, we developed two data pipelines to process extracellular and intracellular signal features. An overview of the extracted features and detailed descriptions can be found in Table 1. Among the extracellular signal features, parameters, such as R-wave spike amplitude, R-wave spike width, and field potential duration (FPD) were used as illustrated in Figure 2A.

**TABLE 1:** Overview of the feature types and names, along with detailed descriptions of the respective computational processes involved in their derivation.

Feature type	Feature name	Description
Extracellular signal features	R_amplitude	Difference between the maximum and minimum voltage values of the R spike
	R_width	Width of the R spike, between the positive and negative peak
	FPD	Distance between the R spike and the T wave signal peak
	conduction_speed	Mean of local conduction speeds computed at each electrode location
	rec_duration	Total recording duration in seconds
	rec_proc_duration	Duration of the processed recording segment
	n_beats	Number of synchronous beats
	n_electrodes_sync	Number of electrodes that captured synchronous activity
	active_area_in_percent	Percentage of electrodes that captured synchronous activity
	mean_nni	Mean of RR-intervals
	sdnn	Standard deviation of RR-intervals
	sdsd	Standard deviation of differences between adjacent RR-intervals
	rmssd	Square root of the mean of the sum of the squared differences between adjacent RR-intervals
	median_nni	Median absolute values of successive differences between RR-intervals
	nni_50	Number of interval differences of successive RR-intervals larger than 50 m
	pnni_50	Proportion derived by dividing nni_50 by the total number of RR-intervals
	nni_20	Number of interval differences of successive RR-intervals larger than 20 m
	pnni_20	Proportion derived by dividing nni_20 by the total number of RR-intervals
	range_nni	Difference between the maximum and minimum RR-intervals
	cvsd	rmssd divided by mean_nni
	cvnni	Ratio of sdnn divided by mean_nni
	mean_hr	Mean heart rate
	max_hr	Maximum heart rate
	min_hr	Minimum heart rate
	std_hr	Standard deviation of the heart rate
Intracellular signal features	AP_amplitude	Difference between the maximum and the lowest dip of the AP wave
	depolarization_time	Time from the beginning of the upstroke until the potential reaches the maximum amplitude
	APD_50_	Time from the beginning of the upstroke until the potential reaches 50% repolarization
	APD_90_	Time from the beginning of the upstroke until the potential reaches 90% repolarization
Recording information	gain	Gain settings used for the recording
	cell_line	Name of the cell line
	compound	Name of the drug, if the recording was made during drug experiments
	file_path	File path where the recording file was stored

The conduction speed was computed following the method described in previous studies (Dunham et al., 2022; Bayly et al., 1998), with some modifications. In brief, the elapsed time, denoted as T, from the onset of wave propagation, along with the x and y coordinates of the electrode within the MEA, were fitted to a three-dimensional, cone-shaped surface (Figure 2C). This representation can be found in Equation 1.

$$T=\sqrt{a{\left(x-x^{\prime }\right)}^{2}+b{\left(y-y^{\prime }\right)}^{2}}+c$$ (1)

In Equation 1, the coefficients are represented as *a*, *b*, and *c*, while $x^{\prime }$ and $y^{\prime }$ are the coordinates of the wave propagation initiation. Following the fitting of the data to the three-dimensional, cone-shaped surface, local conduction velocities at each electrode location were computed as described previously (Bayly et al., 1998). Subsequently, the magnitudes of local conduction velocities were averaged to derive an estimate of the overall conduction speed.

Among other extracellular signal features, time domain heart-rate-variability (HRV) features were computed using a previously published Python package (Champseix et al., 2021). The HRV features include metrics, such as mean values and standard deviations of intervals of consecutive R spikes (RR-intervals) (Electrophysiology, 1996). HRV features and other features, such as intracellular signal features (Figure 2B) and recording information, are detailed in Table 1. Intracellular signal features were extracted from intracellular-like signals recorded by the HD-MEAs. As described in a previous study, three consecutively measured waveforms - recorded either 9 s after electroporation or after channel stabilization - were averaged to obtain a single waveform per channel (Lee et al., 2022). Signals with a peak amplitude larger than 1 mV and a peak width exceeding 50 m were classified as intracellular-like signals. These criteria can be easily adjusted by users to suit their specific needs.

### Cardiomyocyte differentiation and culture

The generation of human iPSCs was approved by the Ethics Committee of the University Medical Center Göttingen and carried out in accordance with the approved guidelines (see Ethics Statement section for details). A human iPSC line UMGi129-A, termed SQT5-line, was derived from a short-QT-syndrome type-5 (SQT5) patient harboring a known variant in the CACNB2 gene. The CACNB2 gene is responsible for encoding L-type Ca^2+^ channels, and variants in this gene have been identified as being linked to short QT syndrome (Garg et al., 2018; El-Battrawy et al., 2021). The SQT5-line’s isogenic control UMGi129-A-1, the SQT5corr-line, was generated after correcting the variant in the CACNB2 gene using ribonucleoprotein-based CRISPR/Cas9. Both the SQT5-line and SQT5corr-line were generated in a prior study (El-Battrawy et al., 2021) and delivered in frozen cryotubes for this research. The iPSC lines were then differentiated into spontaneously beating CMs, following a protocol established in the previous study (El-Battrawy et al., 2021). Additionally, commercially available CMs, differentiated from healthy donor iPSCs and referred to as iCell Cardiomyocytes, were purchased from Fujifilm Cellular Dynamics International (Madison, Wisconsin, United States). The culturing of these cells was performed according to the manufacturer’s guidelines.

### Cell plating and activity measurement on the HD-MEA

To record the activity of CMs, we used HD-MEAs (Ballini et al., 2014), which were equipped with 26,400 microelectrodes and 1,024 readout channels. These HD-MEAs were used to measure both intracellular-like and extracellular signals from CMs (Lee et al., 2022). Intracellular-like signals, obtained through the HD-MEAs after electroporation (Lee et al., 2022), featured action potential (AP) waveforms similar to those recorded by current-clamp patch measurements. In contrast, the signal amplitude was considerably lower (Lee et al., 2022). The shape similarity facilitated the extraction of AP wave features, including the action potential duration (APD). Before plating the CMs, the HD-MEAs underwent sterilization through immersion in 70% ethanol, followed by thorough rinsing with deionized water and drying under a laminar flow hood. The electrode array, with a size of approximately 4 × 2 mm^2^, was prepared by coating it with human fibronectin solution (Cat. FC010, Merck KgaA, Darmstadt, Germany) at a concentration of 50 μg/mL. This coating process involved incubation at 37°C for an hour, providing optimal conditions for cellular adhesion, thereby enhancing the quality of the captured signals.

Following the plating of CMs on the HD-MEAs, the devices were placed in a humidified incubator with 5% CO_2_ to allow for recovery and optimal growth. This environment was maintained for over 7 days until spontaneous beating was observed, indicating successful cellular adaptation and functioning. The cell culture medium was refreshed three times a week to maintain cell health and vitality during measurements. In this study, the term “culture” denotes an ensemble of cells plated on the same HD-MEA chip or in the same well sharing the same culture medium.

The activity measurements on the HD-MEAs were consistently conducted within a humidified incubator set at 37°C with 5% CO_2_. The controlled environment ensured reproducibility and reliability of our cellular activity measurements. For the extracellular measurements, cellular activity was screened over the entire electrode array area using the MaxLive Software (version 19.2.27, MaxWell Biosystems AG, Zurich, Switzerland). Thereafter, 1,020 electrodes featuring the largest signal amplitudes were selected. A similar process was applied to the intracellular measurements, with cellular activity screened and 200 electrodes with the largest signal amplitudes selected. We then performed electroporation on these 200 electrodes to measure intracellular-like signals according to the procedure that has been previously described (Lee et al., 2022) and is also abstracted in Supplementary Method 1.

### Drug testing protocols

Dimethyl sulfoxide (DMSO, Cat. D4540), quinidine (Cat. 22600), nifedipine (N7634), flecainide (Cat. F0120000), amiodarone (Cat. A8423), sotalol (Cat. S0278), and disopyramide (Cat. D2920000) were purchased from Merck KGaA (Darmstadt, Germany). Ivabradine (Cat. HY-B0162A) and ranolazine (Cat. HY-B0280) were purchased from Lucerna-Chem AG (Lucerne, Switzerland). All drug solutions, except ivabradine and sotalol, were prepared in a two-step procedure. The initial dissolution of the compounds was carried out in DMSO, followed by a subsequent dilution in the culture medium. This procedure was specifically designed to ensure that the DMSO concentration remained below 0.1% during drug testing. Ivabradine and sotalol were dissolved directly in the culture medium. For the SQT5-line, drug concentrations in the HD-MEAs were gradually increased using an additive approach to investigate the effects of escalating doses.

We explored the influence of quinidine, flecainide, disopyramide, ivabradine, amiodarone, sotalol, and ranolazine on CMs differentiated from the SQT5-line iPSCs. The baseline recordings, which were performed before the administration of drugs, were measured multiple times in the same culture. This procedure was aimed at ruling out the possibility that the electrophysiological perturbations observed after drug administration were due to intrinsic changes in the cardiomyocytes rather than the effects of the drugs. After baseline measurements, the drug concentration was sequentially increased as specified in Table 2, with each increment separated by 45-minute intervals, unless otherwise specified in respective figures. Extracellular signals were recorded at each concentration level and stored in standard HDF5 format using the MaxLive Software.

**TABLE 2:** List of drugs and their concentrations used in the drug testing experiments.

Recording type	Cell line	Drug	Concentration(s) (µM)
Extracellular recording	SQT5-line	Quinidine	0.1, 0.3, 1, 3, 10
		Flecainide	0.03, 0.1, 0.3, 1, 3
		Disopyramide	3, 13, 43
		Ivabradine	0.3, 1, 3
		Amiodarone	0.03, 0.1, 0.3, 1, 3
		Sotalol	3, 10, 30, 100, 300
		Ranolazine	0.3, 1, 3
Intracellular-like recording	iCell Cardiomyocytes	Nifedipine	0.1
		Quinidine	1
		Sotalol	30

We also investigated the impact of nifedipine, quinidine, and sotalol on the intracellular signals of iCell Cardiomyocytes using electroporation. The applied drug concentrations are specified in Table 2. After establishing three baseline measurements at one-hour intervals, a pre-diluted drug solution was administered to the HD-MEA 30 min after the latest baseline measurement to reach the respective concentration. Intracellular-like signals were subsequently recorded 30 min after the addition of drugs.

## Results and discussion

### Modular and structured data analysis pipeline

We developed a data analysis platform, named “CardioMEA”, using the Kedro framework to process and analyze data collected from CMs on HD-MEAs. The highly modular nature of our data processing pipeline ensures that each step (node) is clear and easy to understand. The code uses the nodes as building blocks arranged in a specific order to generate the pipeline (Kedro framework). All pipelines developed within this study are listed in the file “pipeline_registry.py”. This comprehensive record is intended to provide easy access to each pipeline, simplifying navigation and usage throughout the data analysis.

Details pertaining to input and output data, such as the paths to their respective storage locations and configurations for data loading and saving, are cataloged in a dedicated file, called “catalog.yml”. This arrangement is particularly advantageous, as it simplifies the management of inputs and outputs without the need to navigate through the entire code.

CardioMEA is publicly accessible on GitHub via the following link: https://github.com/leejheth/CardioMEA. To use CardioMEA, the initial requirement is to install GNU make, a program that simplifies the execution of command sets. Setting up the working environment is straightforward, requiring only the execution of the ‘make setup’ command in the terminal. This command will automatically generate a new virtual environment and install all necessary dependencies within it. Additionally, the repository includes a troubleshooting guide to assist researchers in setting up the virtual environment, as well as in processing and visualizing the data. This user-friendly setup ensures easy access to our sophisticated data-processing tools for a broad community of researchers.

### Feature extraction from multiple data files in parallel

Experiments often result in a multitude of recording files requiring specific processing. Undertaking this task for each file can be cumbersome and time-consuming. Therefore, we designed CardioMEA with the capability to handle and process multiple data files concurrently, using multiple CPUs. Users can predetermine the number of CPUs to scale the process, depending on the resource availability of their workstations or high-performance computing clusters.

Initially, the user needs to supply a CSV file containing a list of directories housing the recording files, along with corresponding cell line names, compounds (drugs), and additional notes as applicable. These notes may include any relevant experiment details, such as compound concentrations or experiment IDs. Subsequently, by executing the “create_list” pipeline, a complete list of recording files, stored in the specified directories, can be identified and listed in a newly created CSV file. The recording files, listed in this CSV file, are then processed in batches, with the batch size being equivalent to the number of CPUs predetermined by the user. This approach allows for efficient parallel processing and significantly reduces the time required to process large data volumes, as compared to previously published data analysis platforms that process data sequentially (Supplementary Figure S1).

**FIGURE 3:**
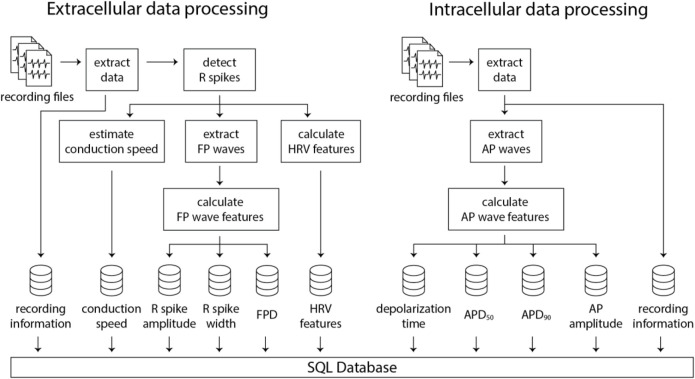
Illustration of the data processing pipelines for extracellular and intracellular data obtained using HD-MEAs. Numerous recording files can be processed in parallel using multiple CPUs, which significantly decreases computation time and enhances efficiency.

The subsequent stage involves feature extraction from the recording files. The feature extraction pipelines for extracellular and intracellular data are illustrated in Figure 3. The information for the extracellular data is extracted from the recording file, followed by identifying the spike time points. Then, FP wave features, conduction speed, and HRV features are computed. Similarly, intracellular data extraction occurs from the recording file, which is then followed by the calculation of AP wave features. All extracted feature values, coupled with a timestamp indicating when the processing was completed, are uploaded to a PostgreSQL database. Two distinct SQL tables are utilized, each for extracellular and intracellular data. Each processing result is inserted as a row in the SQL table accompanied by a timestamp, allowing for comprehensive data history preservation within the database. This feature proves particularly beneficial in scenarios where processing steps need to be modified or altered. It enables the tracking of previously processed data, thereby ensuring traceability of all data transformations.

The authors of Cardio PyMEA - a previously published Python-based platform - reported a performance issue, i.e., that the graphical user interface would freeze during data processing due to Python’s global interpreter lock (GIL). As demonstrated in this study, we effectively addressed this problem in CardioMEA by separating the data processing and visualization pipelines and by using a database to store the processed data. Additionally, we resolved the data processing speed issue noted by Cardio PyMEA’s authors by implementing multiprocessing in CardioMEA, which allows for parallelized data processing. Table 3 includes a comparison of open-source data analysis platforms for cardiac data obtained from MEAs.

**TABLE 3:** Comparison of open-source data analysis platforms for cardiac data obtained through MEAs.

	CardioMDA (Pradhapan et al., 2013)	MultiElec (Georgiadis et al., 2015)	Cardio PyMEA (Dunham et al., 2022)	CardioMEA (this study)
Programming language	MATLAB	MATLAB	Python	Python
Graphical user interface (GUI)	MATLAB-based GUI	MATLAB-based GUI	Python-based GUI	Web-based GUI
Execution of data processing pipelines	Sequential	Sequential	Sequential	Parallel
Comparison of different experiment conditions (i.e., cell lines, drug concentrations) in the GUI	Available	Not available	Not available	Available
Wave propagation analysis	Not available	Available	Available	Available
Intracellular-like data analysis	Not available	Not available	Not available	Available
Data processing and GUI	Single pipeline	Single pipeline	Single pipeline	Split into separate pipelines

### Feature correlations between different types of HD-MEA data and whole-cell current-clamp patch measurement data

**FIGURE 4:**
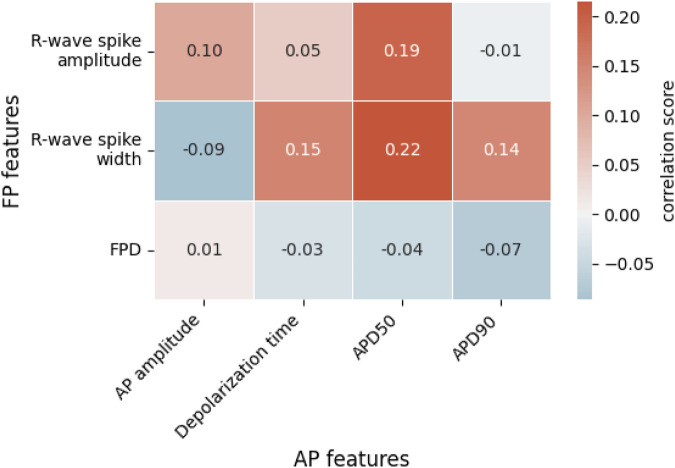
Correlations between extracellular field potential (FP) and intracellular-like action potential (AP) features. Each value represents the Spearman rank correlation coefficient. FPD, field potential duration; APD50, action potential duration at 50% repolarization; APD90, action potential duration at 90% repolarization.

As CardioMEA cannot only process extracellular signals but also intracellular-like signals from iPSC-derived CMs, researchers can study the relationship between these two types of electrical signals (extracellular and intracellular-like) recorded by the very same electrodes and originating from the same cells. Such an analysis offers a unique perspective on the relationship between extracellular and intracellular or intracellular-like signal and waveform features. We conducted a correlation analysis between features derived from the two different data types. Utilizing data from 29 CM cultures (iCell Cardiomyocytes), we analyzed signals obtained from a total of 3,987 electrodes, which captured both extracellular and intracellular-like signals. Figure 4 presents the correlations between features extracted from the two signal types.

In Figure 4, we see weak positive correlations between R-wave spike width and depolarization time, APD_50_, and APD_90_. The R-wave spike appears in the depolarization phase of an AP, which supports a correlation between the R-wave spike width and the AP’s depolarization time. Both APD_50_ and APD_90_ are measured from the start of depolarization and are, therefore, connected to the R-wave spike width. Among the FP features shown in Figure 4, the R-wave spike amplitude is the only feature expressed in terms of voltage; in contrast, R-wave spike width and FPD are time-based measures. Regarding the R-wave spike amplitude, we noticed that R-wave spikes were clipped in some channels due to their high signal magnitude. While the gain may be reduced during recordings to address this clipping issue, it entails the risk of diminishing the T-wave amplitude, which is already small. Consequently, the R-wave spike amplitude is not an ideal feature for correlation analysis.

Interestingly, FPD did not strongly correlate with either APD_50_ or APD_90_, even though we anticipated a relationship with these two parameters. In fact, FPD, in our correlation analysis, showed minimal correlation with all AP-related features. This could be attributed to the noise levels in the FPD data that occasionally obscured the detection of the T-wave. We noted that the T-wave and its exact timing often could not be precisely determined, especially for extracellular signals of iCell Cardiomyocytes featuring very small T-wave amplitudes. Therefore, extracting FPD values and calculating their correlations to other features is challenging.

**FIGURE 5:**
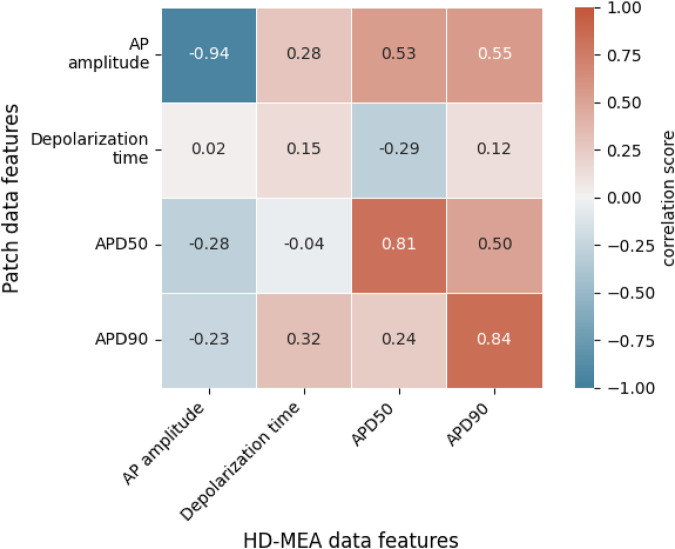
Correlations between intracellular features obtained with patch clamp measurements in current-clamp mode and features of intracellular-like data obtained by HD-MEAs in simultaneous measurements from the same cells. A total of 83 AP waveforms collected from 2 cells (cell A, cell B) were used to compute the correlation scores. Each value represents the Spearman rank correlation coefficient. APD50, action potential duration at 50% repolarization; APD90, action potential duration at 90% repolarization.

Next, we derived intracellular-like features from HD-MEA data and whole-cell current clamp patch data to examine the correlation between the respective feature values. For this analysis, we used recordings by patch clamp and intracellular-like recordings by the HD-MEA, which were obtained simultaneously from the same cells. The data were published (Lee et al., 2022) in a previous study (cell A, Supplementary Figure S2A; cell B; Supplementary Figure S2B). Figure 5 shows the correlation between features extracted from 60 AP waveforms over a span of 124 s (cell A) and from 23 AP waveforms over a span of 36 s (cell B) that have been captured through simultaneous patch clamp and HD-MEA recordings.

**FIGURE 6:**
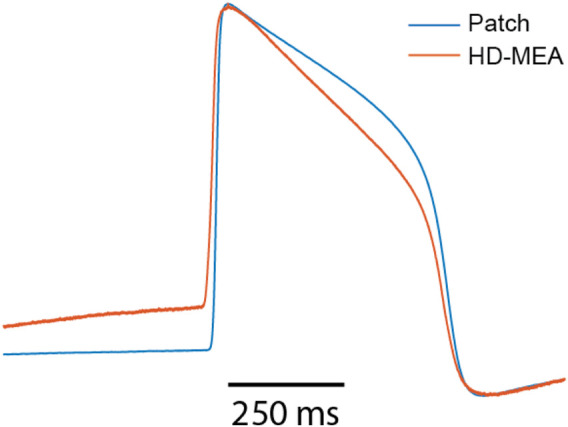
Overlay of amplitude-normalized AP waveforms, averaged across 60 consecutively recorded waveforms (cell A) and obtained from patch-clamp and HD-MEA measurements.

Figure 5 shows that AP amplitudes of the patch clamp and the HD-MEA data are negatively correlated. This negative correlation is, however, a consequence of the fact that both signals are simultaneously measured from the same cell(s). As discussed in our previous study (Lee et al., 2022), ion and current leakage through nanopores, which are generated transiently by the electroporation, reduce the patch clamp signal. The poration-induced leakage decreases over time as the cell membrane reseals. The resealing of the cell membrane increases the AP amplitude of the patch clamp recording, while the AP amplitude in the HD-MEA measurement is concurrently decreasing (Supplementary Figure S2). APD_50_ and APD_90_ exhibited strong positive correlations between patch clamp and HD-MEA measurements, indicating that the two recording methods capture consistent APD patterns over time and while the cell membrane reseals. Depolarization times, on the other hand, were only weakly correlated between the two recording methods. This finding may be due to differences in waveform shapes, as shown in Supplementary Figure S2, Figure 6. In particular, the AP waveforms, captured by HD-MEA measurements, exhibit a gradual increase of the voltage before the rapid upstroke (before Phase 0) which is much less pronounced in the AP waveform captured by the patch clamp measurement. As the data are collected simultaneously from the same cells, this marked difference may be attributed to the difference in the two recording settings, most prominently the electrode configuration (penetrating patch pipette and outside Pt-black coated planar electrode).

Interestingly, the AP waveforms captured by the HD-MEA closely resemble those from previous studies (Hayes et al., 2019; Jans et al., 2017; Edwards et al., 2018) that reported electroporation-mediated intracellular-like recordings with planar electrodes on MEAs. Conversely, when sharp 3D nanostructures were employed for intracellular-like measurements, AP waveforms obtained through MEAs more closely resembled those of patch clamp measurements (Jahed et al., 2022; Lin et al., 2017). These observations suggest that the difference in AP waveform shapes between patch-clamp and HD-MEA recordings may be attributed to differences in electrode shape and arrangement, which likely influence the correlation between depolarization time values (Figure 5) obtained from both methods.

As becoming evident from this section, CardioMEA’s unique features and unprecedented capability of processing and analyzing both extracellular and intracellular-like signals, captured by HD-MEAs, offer invaluable insights. These insights enhance our understanding of cardiac electrophysiology and facilitate correlation analyses between different data types.

### Interactive dashboard for exploratory data analysis and visualization

After data processing, the availability of an interactive tool that enables scientists to visualize the processed data and conduct exploratory data analysis is essential for data-driven analysis. Therefore, we have incorporated a web-based interactive dashboard within CardioMEA, termed “CardioMEA Dashboard”. This user-friendly interface facilitates the visualization of data in the SQL database, further investigations of features, and downloading the resulting figures. Furthermore, the web-based nature of the CardioMEA Dashboard makes it a very accessible tool. Unlike traditional GUI-based software, the CardioMEA Dashboard does not require specific installation, thereby offering enhanced compatibility with a broad range of user operating systems. This feature ensures universal applicability and ease of use, rendering the CardioMEA Dashboard a reliable and convenient tool for biomedical researchers working in diverse computational environments.

**FIGURE 7:**
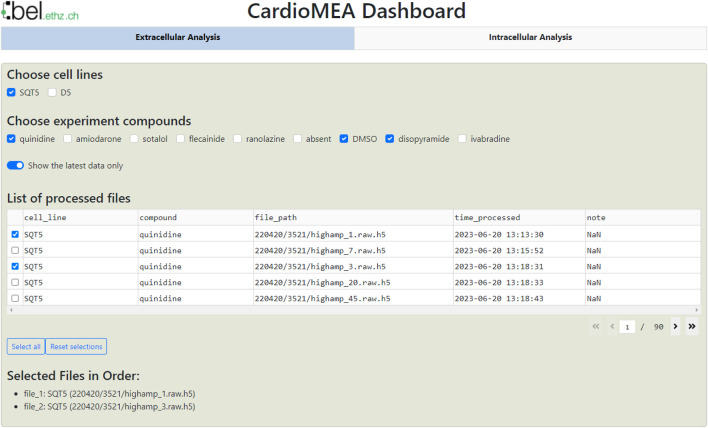
CardioMEA Dashboard data panel. The cell lines stored in the SQL database appear at the top row of the data panel, while the compounds, used with each selected cell line, are shown in the subsequent row. Users have the option to display either all historical processed data or limit the display to the most recent data. Based on these settings, processed files are listed in the table, which allows for selecting specific data for visualization and further analysis.

The dashboard’s data panel, illustrated in Figure 7, displays all cell lines and compounds found in the existing SQL database, either from the extracellular or intracellular data table. Upon choosing specific cell lines and compounds, data corresponding to the selected criteria will be presented in the ‘List of processed files’ table. Users can then select multiple files of interest, which will be displayed at the bottom of the data panel. This arrangement empowers users to navigate the SQL database and interactively select data for analysis without the need of proficiency in SQL.

**FIGURE 8:**
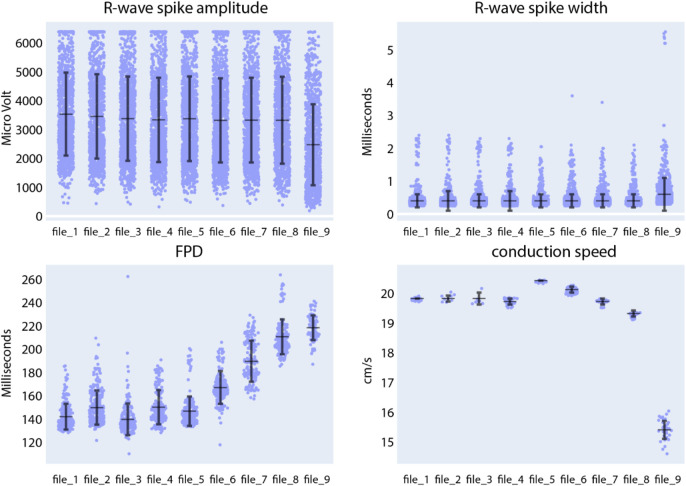
Compound analysis of signals of CMs derived from SQT5-line iPSCs using the Extracellular Analysis panel. Following four initial baseline measurements (file_1 to file_4), the concentration of quinidine was sequentially increased in the following sequence: 0.1 µM (file_5), 0.3 µM (file_6), 1 µM (file_7), 3 µM (file_8), 10 µM (file_9). Each data point in the provided figures corresponds to a value obtained from a single recording electrode. The horizontal and vertical bars denote the mean values and standard deviations (mean ± standard deviation), respectively. In the R-wave spike amplitude plot, the clipped data points represent 6.4% of the data in the first baseline measurement.

Located beneath the data panel is a visualization and analysis panel divided into three tabs (see also Supplementary Figure S11). The first tab, titled “Data distribution”, contains a set of figures visualizing the data. As an example for extracellular data analysis, Figure 8 represents the evolution of the R-wave spike amplitude, the R-wave spike width, FPD, and the conduction speed during an increment of quinidine concentration dosed to CMs, differentiated from SQT5-line iPSCs, after four baseline measurements. Quinidine is a Class Ia antiarrhythmic drug that is known to prolong cardiac repolarization (Zwi et al., 2009; Millard et al., 2018). In clinical investigations involving short-QT-syndrome patients, quinidine effectively prolonged the QT interval (Wolpert et al., 2005; Gaita et al., 2004). Other studies have reported that quinidine prolonged APDs of CMs derived from short-QT-syndrome type-1 patients (El-Battrawy et al., 2018; Shinnawi et al., 2019). When quinidine was applied to CMs derived from an SQT5 patient, we observed an increase in FPD with increasing quinidine concentration (Figure 8), which is in agreement with findings from a previous study (El-Battrawy et al., 2021), suggesting that quinidine may also effectively prolong the QT interval of SQT5 patients. Additionally, a decrease in signal-conduction speed was observed as the quinidine concentration increased. This reduction in conduction velocity can be attributed to quinidine’s blocking effect on Na^+^ channels. By limiting the influx of Na^+^ ions into the cell, quinidine may indirectly slow down the diffusion of Na^+^ ions to adjacent cells that are interconnected through gap junctions.

We subsequently assessed the responses of SQT5-line CMs, subjected to other drugs listed in Table 2, while focusing on the drugs’ efficacy in prolonging the FPD. Both compounds, disopyramide (Supplementary Figure S3) and sotalol (Supplementary Figure S4), caused a dose-dependent prolongation of FPDs in CMs derived from SQT5 patients. On the other hand, flecainide (Supplementary Figure S5), ivabradine (Supplementary Figure S6), amiodarone (Supplementary Figure S7), and ranolazine (Supplementary Figure S8) did not or only minimally affect the FPD. These observations indicate that disopyramide and sotalol could be explored as alternative therapeutic options for treating arrhythmias in SQT5 patients, especially when quinidine proves ineffective or is unavailable.

**FIGURE 9:**
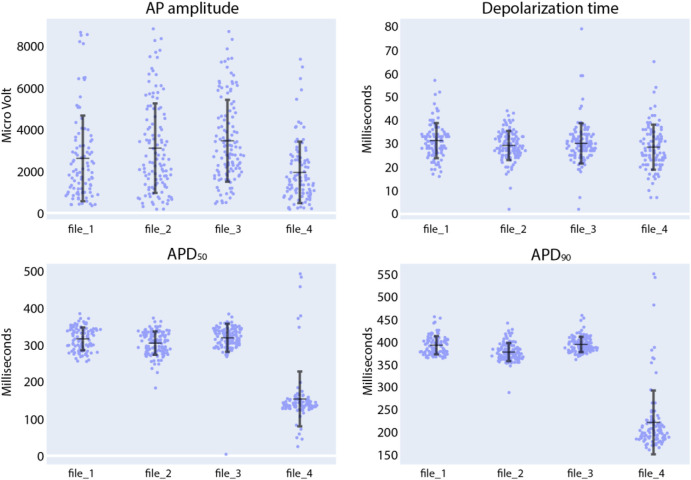
Compound analysis of signals of iCell Cardiomyocytes using the Intracellular Analysis panel. Following three initial baseline measurements (file_1 to file_3), nifedipine was added to the culture to reach a concentration of 100 nM (file_4). Each data point in the provided figures corresponds to a value obtained from a single recording electrode. The horizontal and vertical bars denote the mean value and standard deviation (mean ± standard deviation), respectively.

Next, in the Intracellular Analysis panel of the CardioMEA Dashboard, we delved into the drug-induced modulations observed in the features of intracellular-like signals. Figure 9 illustrates the evolution of AP amplitude, depolarization time, APD_50_, and APD_90_ measured from iCell Cardiomyocytes, when the cells were exposed to nifedipine following three baseline measurements. Nifedipine is a Ca^2+^ channel blocker that shortens APDs (Scheel et al., 2014). As anticipated, nifedipine did not result in any substantial alteration of the AP amplitude or depolarization time. However, it significantly shortened APD_50_ and APD_90_, as evident from the visualization panel. A reduction in Ca^2+^ current renders the K^+^ current dominating during cardiac repolarization, leading to an abbreviated APD.

We further explored the effects of quinidine and sotalol on the AP features of iCell Cardiomyocytes. As shown in Supplementary Figure S9, exposure of CMs to 1 µM of quinidine increased depolarization time and APD_90_. The Na^+^ channel-blocking capability of quinidine and its effects on FPD prolongation have been previously documented (Zwi et al., 2009; Millard et al., 2018). When subjected to 30 µM of sotalol (Supplementary Figure S10), there was negligible change in depolarization time (Dobrev et al., 2012), but we noted a notable rise in both APD_50_ and APD_90_. These observations are consistent with sotalol’s properties as a Class III, K^+^ channel blocker. As demonstrated by the analysis of the effects of seven clinically used drugs on extracellular signals and of three drugs on intracellular-like signals, CardioMEA holds promise as a versatile platform for data analysis and visualization in the field of MEA-based drug testing.

The second tab of the visualization and analysis panel, ‘Recording Info’, displays a table showing recording information and other features of the selected data files (Supplementary Figure S11). The third tab, exclusively available for the Extracellular Analysis panel and titled ‘Feature Analysis’, will be discussed in the subsequent section.

### Feature analysis using automated machine learning

To identify key electrophysiological phenotypes and disease biomarkers that can be used to distinguish diseased cell lines from healthy controls, it is crucial to determine which features have a high predictive potential. To address this point, we incorporated automated machine learning (AutoML) and feature-importance analysis tools into CardioMEA, enabling users to run these complex analyses through the dashboard without the need to write any code.

**FIGURE 10:**
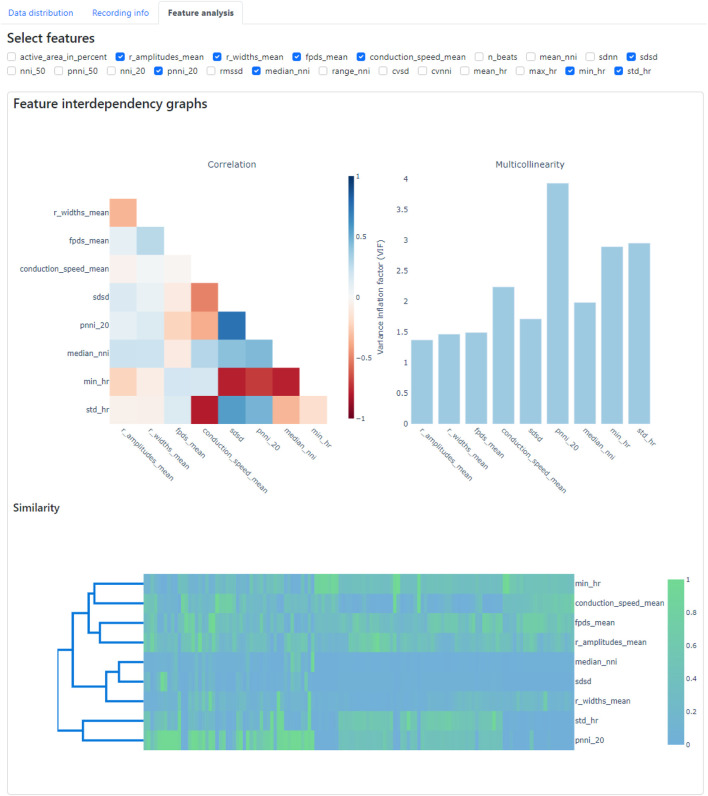
Feature analysis tab within the CardioMEA Dashboard, showing feature interdependency graphs. The correlation, multicollinearity, and similarity between selected features were computed and displayed.

In the Feature Analysis tab, users can select a subset of features from the data selected in the data panel with the assistance of a correlation heatmap, multicollinearity plot, and similarity cluster map (shown in Figure 10). Numerous features are extracted during the feature extraction process, and it is crucial to select a subset of these features before constructing classification models to discern diseased and healthy cell lines. This selection process is important, because some features may be highly correlated, exhibit high collinearity, or may be similar to each other, which may potentially interfere with the feature importance analysis. For example, permutation analysis (Breiman, 2001), one of the essential techniques for investigating feature importance (Petch et al., 2022), could be affected, as collinear or similar features could compensate for permuting a feature. As a result, classification performance may not decline when one of the collinear features is permuted.

To demonstrate the usage of Feature Analysis, we selected recording data from all cultures without drug administration in the data panel to investigate which features are crucial in distinguishing SQT5-line and SQT5corr-line CMs. In total, 93 cultures of SQT5-line CMs and 33 cultures of SQT5corr-line CMs were included for feature importance analysis.

The criteria or thresholds for eliminating redundant features in CardioMEA are customizable by the user, allowing for flexibility based on specific needs or applications. In this study, we demonstrated feature elimination by jointly assessing correlations, multicollinearity, and feature similarity. Specifically, features were removed if they exhibited absolute correlation coefficients greater than 0.8, multicollinearity indices exceeding 5, or high similarity as determined through visual inspection on the dashboard (Figure 10). Users can adjust these thresholds according to their domain knowledge and the particular requirements of their disease or drug-related analyses. The CardioMEA Dashboard’s interactive plots update in real-time, which enables users to immediately observe the effects of feature selection on correlation, multicollinearity, and similarity metrics. This dynamic feedback supports informed decision-making during feature selection. Figure 10 presents a screenshot of the dashboard showing the selected 9 features after eliminating redundant features using a correlation heatmap, multicollinearity plot, and similarity cluster map.

**FIGURE 11:**
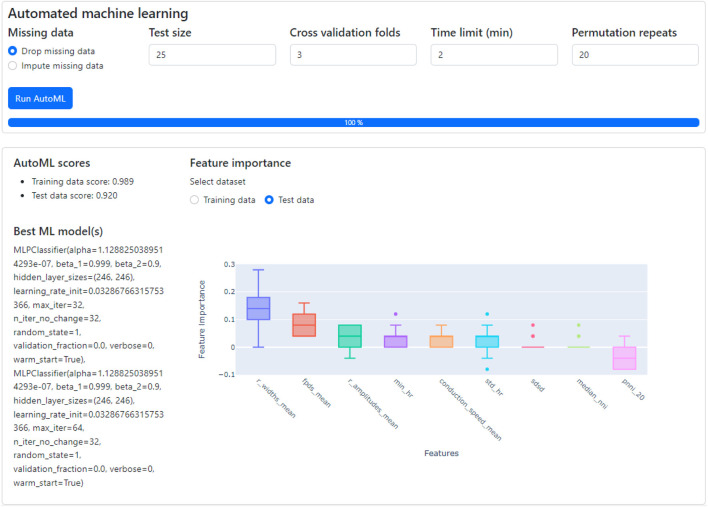
The classification of CMs derived from SQT5-line and SQT5corr-line iPSCs, followed by an analysis of feature importance. With just a few mouse clicks, users are empowered to perform automated machine learning to build the optimal classification model. The performance and details of the optimized model are displayed in the lower left section, along with the outcomes of a feature importance analysis (lower right section). Given the respective ML model, this analysis shows the predictive power of selected features for distinguishing between SQT5-line and SQT5corr-line CMs.

After the selection of features, the ensuing step involves investigating their importance. Within CardioMEA, we have integrated an AutoML functionality to identify the optimal model for classification purposes. This functionality has been realized by harnessing the AUTO-SKLEARN toolkit (Feurer et al., 2015), an open-source library that facilitates an efficient and automated approach to machine learning model selection and hyperparameter tuning (Supplementary Method 2). The user is empowered to determine how missing data is handled and to set the test data size, the number of cross-validation folds, the time limit per fold, and the number of permutation repeats (Figure 11). By simply clicking ‘Run AutoML’, the AutoML algorithm is triggered.

There are several risk factors to consider when applying machine learning in data analysis. The best-known risk is overfitting, which occurs when a model has been extensively fitted to the training dataset, leading to poor generalization on unseen data (test data). To address this issue, the AutoML pipeline used in this study incorporates cross-validation, ensuring that the model has been evaluated on different subsets of the data to better estimate its generalizability. Additionally, although AUTO-SKLEARN offers the option to build an ensemble of top-performing models to enhance performance, we disabled this feature to avoid overfitting, as complex models are more susceptible to overfitting. Another issue in using machine learning is the “black box” nature of many models, which can make interpretation difficult. To improve model interpretability, we implemented feature permutation analysis, a technique that assesses the contribution of each feature to the model’s performance.

As shown in the lower left section of Figure 11, the AutoML process yielded the result that the multi-layer perceptron (MLP) classifier delivered the best performance for the provided dataset, applying a 3-fold, stratified cross-validation (sCV) method. This model achieved a classification accuracy of 98.9% for the training dataset and 92.0% for the test dataset. We compared this result with the outcomes of a baseline model, which classified the data based on the most frequent labels. Upon conducting this procedure 10 times with a 5-fold sCV approach, the baseline model yielded an accuracy of 71.8% ± 1.8% (mean ± standard deviation). Next, employing the MLP classifier with its default parameters in the SKLEARN library (Pedregosa et al., 2011) yielded 80.1% ± 5.3% accuracy. The comparison between the baseline model and the default MLP classifier with the model constructed by AutoML shows that AutoML identifies and fine-tunes an optimal model, thereby enhancing performance.

Subsequently, CardioMEA computes feature importance values using the optimized model. This process involves a permutation of the values of each feature individually and gauging the subsequent decline in accuracy over a predefined set of iterations (Breiman, 2001). As depicted in Figure 11 in the lower right section, R-wave spike width and FPD were identified as the top two significant features. This finding suggests that these features play a pivotal role in the classification of SQT5-line and SQT5corr-line cells when utilizing the AutoML-trained model. It is crucial, however, to emphasize that permutation importance indicates the significance of a specific feature for a particular model, which is the MLP classifier in this case, rather than representing its innate predictive power. As demonstrated in the context of Figure 11, users can efficiently construct optimal machine learning models using the dashboard, bypassing the need to manually code the algorithms, which significantly simplifies the process of identifying critical features for distinguishing between diseased cell lines and healthy controls.

Feature permutation analysis includes to randomly vary the values of one feature and observing the change in model performance. A significant performance drop indicates that the respective feature is important, while little or no drop suggests that the feature is less important. However, due to the stochastic nature of the process, this method inherently introduces variability.

Feature permutation analysis, as shown in Figure 11, exhibits a few outliers (dots) in several features, such as min_hr, std_hr, sdsd, and median_nni. One reason for the outliers could be the presence of strong correlations between multiple features. When one feature is permuted, this permutation can upset the respective correlations as only the values of a specific feature are varied, which then may lead to a significant accuracy drop. In this case, an outlier may indicate a correlation effect rather than reflecting the feature’s individual contribution. The outliers and high variance may also indicate that the model is sensitive to certain features or subsets of the data. If the data distribution is skewed or a feature is important for only a subset of observations, permuting that feature could lead to larger-than-expected performance drops.

To reduce the impact of outliers, repeating the permutation analysis multiple times may help to smooth out random fluctuations of the feature importance score. The number of repeats is configurable by the user (“Permutation repeats” field in Figure 11). This approach could help to reduce the impact of outliers and give a more stable estimate of feature importance.

The aforementioned analysis can be conducted on the dashboard with just a few mouse clicks. Users do not need to delve into the intricate details of constructing machine learning models or analyzing feature importance when exploring their HD-MEA data collected from CMs. This feature demonstrates that the CardioMEA Dashboard can serve as a user-friendly, “no-code” platform for advanced data analysis and visualization.

**FIGURE 12:**
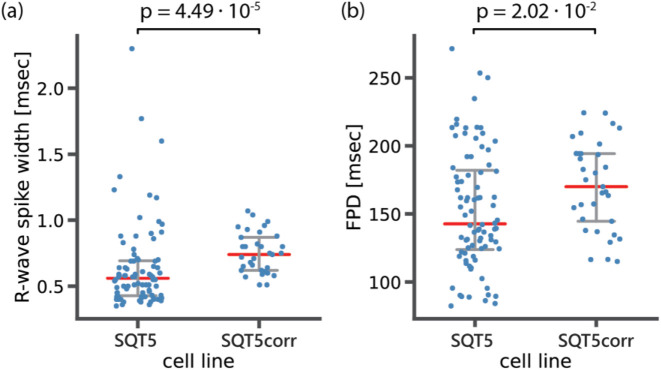
Statistical analysis to compare **(A)** R-wave spike width and **(B)** field potential duration (FPD) between diseased (SQT5) and healthy control (SQT5corr) cell lines. Each data point (blue color) represents one CM culture. Red bars indicate median values and error bars (grey color) indicate interquartile ranges. The p values were estimated using a two-sided Mann-Whitney U test.

We further investigated two features, which emerged as the top two determinants in distinguishing between diseased and healthy cell lines in our feature importance analysis, the R-wave spike width and FPD. Given that the SQT5corr-line was generated by correcting the CACNB2 gene of SQT5, the feature analysis suggests that the mutation within the CACNB2 gene of the SQT5 cells may alter the R-wave spike width and FPD values. A previous study (El-Battrawy et al., 2021) revealed that CMs, differentiated from the same SQT5-line iPSCs, exhibited decreased Na^+^ and L-type Ca^2+^ channel currents. Figure 12 shows a statistical comparison of these feature values - R-wave spike width and FPD - between SQT5-line and SQT5corr-line CM cultures.

As shown in Figure 12A, the observed median value of the R-wave spike width was lower in SQT5 cultures than in SQT5corr cultures. We had anticipated that the R-wave spike width would be larger in SQT5 than in SQT5corr cultures, given that reduced I_Na_ should result in a slower depolarization. However, the range of measured data points obtained from SQT5 cultures is significantly larger than that of SQT5corr cultures. The large spread observed within the SQT5 cultures in Figure 12 suggests substantial variability among the cardiac cultures. This large spread of data points makes it difficult to draw solid conclusions from statistical analysis. Although the statistical analysis did not provide conclusive insights into the relationship of R-wave spike width values between the SQT5 and SQT5corr cultures, the feature importance analysis, carried out via the CardioMEA Dashboard (Figure 11), indicated that the R-wave spike width had the strongest impact on model performance upon permutation. This finding suggests that the machine learning model may identify patterns and interactions that conventional statistical methods may have overlooked. Moreover, it evidences the potentially significant role of the R-wave spike width in distinguishing SQT5 cultures from their isogenic controls.

From a biological perspective, the width of the R spike is associated with the duration of cardiac depolarization. In SQT5 cultures, the heightened importance of the R-wave spike width suggested that alterations in the duration of depolarization could be a key characteristic of the disease in the *in vitro* model. The R-wave spike width showed substantial variance, which rendered it challenging to achieve statistical significance by using traditional methods. Nevertheless, the feature importance analysis of the machine learning approach evaluated each feature’s contribution to the model’s predictive accuracy also considering intricate interactions between features.

The distinction between statistical test and feature importance evidenced the complementary roles of traditional statistical analysis and machine learning approaches. Unlike traditional statistical comparisons, the machine learning algorithm - an MLP classifier in this case - utilized all provided features collectively to distinguish between SQT5 and SQT5corr cultures. The results of the feature analysis evidenced the potential of CardioMEA’s AutoML-driven module to capture complex patterns in the data. This module could improve the identification of novel disease phenotypes, an area where standalone statistical analysis may fall short.

As shown in Figure 12B, FPD values were lower in the SQT5 cultures compared to the SQT5corr cultures. Given the established understanding that mutations in the CACNB2 gene in the SQT5 cell line induce a loss of function in L-type Ca^2+^ channels (El-Battrawy et al., 2021), this decrease of FPD in SQT5 cultures was anticipated. Therefore, the FPD was identified as the second most influential feature in identifying SQT5 cultures, yielding a statistically significant difference between the SQT5 and SQT5corr cultures.

## Conclusion

The study of therapeutic efficacy and potential cardiotoxicity of drugs *in vitro* is a crucial step of preclinical research. HD-MEAs, with their high SNR and spatiotemporal resolution, are instrumental in characterizing the electrophysiology of iPSC-derived CMs. However, until now, there has been a lack of open-source data analysis platforms that can cope with the large data volumes generated by HD-MEA technology.

In this study, we presented CardioMEA, a comprehensive data analysis platform with the ability to process and analyze large volumes of CM data generated by HD-MEAs. CardioMEA offers a complete analysis workflow, from data extraction to exploratory analysis. The platform’s first component provides scalable data processing for feature extraction across multiple data files, while the second component consists of a user-friendly, interactive web-based dashboard for advanced data visualization and analysis. Using CardioMEA, we examined the efficacy of seven clinically used drugs on CMs derived from an SQT5 patient and the cardiotoxicity of three drugs on healthy-donor-derived CMs.

The feature analysis tool within the CardioMEA Dashboard assists users in feature selection and helps to reduce redundancy and multicollinearity in the data. Furthermore, AutoML and feature importance analysis provide insights into the predictive power of features for discerning electrophysiological signatures of diseased and healthy CMs. CardioMEA allows users to perform complex analyses on their HD-MEA data without extensive coding knowledge.

Another unique feature of CardioMEA is its ability to process intracellular signals captured by HD-MEAs, which renders CardioMEA the first of its kind among open-source platforms. The possibility to process both extracellular and intracellular signals renders the platform amenable to a wide range of researchers’ potential needs. CardioMEA offers the possibility to look at the data distribution on the recording electrodes. For statistical analysis, however, it is recommended to use electrode data from several wells or HD-MEAs (biological replicates) to obtain meaningful results.

For future studies, CardioMEA can be augmented by integrating additional modules for performing more detailed analyses of the waveform shape (e.g., T-wave) and its features, or by adding modules for processing and visualizing data obtained from cardiac 3D models, such as cardiac spheroids or engineered cardiac tissues. These 3D models offer a physiologically more relevant representation of the heart compared to traditional monolayer cultures, thereby overcoming some of their inherent limitations. Advances and new developments will be crucial for improving the accuracy of disease modeling and the efficacy of drug testing.

In summary, CardioMEA constitutes a comprehensive and user-friendly platform that significantly advances the analysis of CM signals obtained by using HD-MEAs. The combination of parallel data processing, interactive dashboard, and feature analysis tools enables users to explore and analyze their data efficiently. The platform is expected to advance studies of cardiac diseases and drug testing and to democratize associated high-level data analysis.

## Data Availability

The datasets presented in this study can be found in online repositories. The names of the repository/repositories and accession number(s) can be found below: Zenodo; https://doi.org/10.5281/zenodo.13839126; https://doi.org/10.5281/zenodo.13839556.

## References

[B1] Abbott J. Ye T. Qin L. Jorgolli M. Gertner R. S. Ham D. (2017). CMOS nanoelectrode array for all-electrical intracellular electrophysiological imaging. Nat. Nanotechnol. 12 (5), 460–466. 10.1038/nnano.2017.3 28192391

[B2] Alam S. (2024). Kedro. Available at: https://github.com/kedro-org/kedro (Accessed March 08, 2023).

[B3] Ballini M. Müller J. Livi P. Chen Y. Frey U. Stettler A. (2014). A 1024-channel CMOS microelectrode array with 26,400 electrodes for recording and stimulation of electrogenic cells *in vitro* . IEEE J. Solid-State Circuits 49 (11), 2705–2719. 10.1109/JSSC.2014.2359219 28502989 PMC5424881

[B4] Bayly P. V. Kenknight B. H. Rogers J. M. Hillsley R. E. Ideker R. E. Smith W. M. (1998). Adaptive AR modeling of nonstationary time series by means of Kalman filtering. IEEE Trans. Biomed. Eng. 45 (5), 553–562. 10.1109/10.668741 9581053

[B5] Blinova K. Dang Q. Millard D. Smith G. Pierson J. Guo L. (2018). International multisite study of human-induced pluripotent stem cell-derived cardiomyocytes for drug proarrhythmic potential assessment. Cell. Rep. 24 (13), 3582–3592. 10.1016/j.celrep.2018.08.079 30257217 PMC6226030

[B6] Breiman L. (2001). Random forests. Mach. Learn. 45 (1), 5–32. 10.1023/a:1010933404324

[B7] Champseix R. Ribiere L. Le Couedic C. (2021). A Python package for heart rate variability analysis and signal preprocessing. J. Open Res. Softw. 9 (1), 28. 10.5334/jors.305

[B9] Dobrev D. Singh B. N. (2012). “Antiarrhythmic drugs,” in Electrophysiological disorders of the heart Editors Saksena S. Camm A. J. Second Edition (Philadelphia: W.B. Saunders), 1133–1157. ch. Chapter 80.

[B10] Dragas J. Viswam V. Shadmani A. Chen Y. Bounik R. Stettler A. (2017). A multi-functional microelectrode array featuring 59760 electrodes, 2048 electrophysiology channels, stimulation, impedance measurement and neurotransmitter detection channels. IEEE J. Solid-State Circuits 52 (6), 1576–1590. 10.1109/JSSC.2017.2686580 28579632 PMC5447818

[B11] Dunham C. S. Mackenzie M. E. Nakano H. Kim A. R. Nakano A. Stieg A. Z. (2022). Cardio PyMEA: a user-friendly, open-source Python application for cardiomyocyte microelectrode array analysis. PLOS ONE 17 (5), e0266647. 10.1371/JOURNAL.PONE.0266647 35617323 PMC9135279

[B12] Edwards S. L. Zlochiver V. Conrad D. B. Vaidyanathan R. Valiquette A. M. Joshi-Mukherjee R. (2018). A multiwell cardiac μGMEA platform for action potential recordings from human iPSC-derived cardiomyocyte constructs. Stem Cell. Rep. 11 (2), 522–536. 10.1016/j.stemcr.2018.06.016 PMC6092761 30033088

[B13] El-Battrawy I. Lan H. Cyganek L. Maywald L. Zhong R. Zhang F. (2021). Deciphering the pathogenic role of a variant with uncertain significance for short QT and Brugada syndromes using gene-edited human-induced pluripotent stem cell-derived cardiomyocytes and preclinical drug screening. Clin. Transl. Med. 11 (12), e646. 10.1002/ctm2.646 34954893 PMC8710296

[B14] El-Battrawy I. Lan H. Cyganek L. Zhao Z. Li X. Buljubasic F. (2018). Modeling short QT syndrome using human-induced pluripotent stem cell-derived cardiomyocytes. J. Am. Heart Assoc. 7 (7), e007394. 10.1161/JAHA.117.007394 29574456 PMC5907581

[B15] Electrophysiology T. F. o. t. E. S. o. C. t. N. A. S. o. P. (1996). Heart rate variability. Circulation 93 (5), 1043–1065. 10.1161/01.CIR.93.5.1043 8598068

[B16] Emmenegger V. Obien M. E. J. Franke F. Hierlemann A. (2019). Technologies to study action potential propagation with a focus on HD-MEAs. Front. Cell. Neurosci. 13, 159. 10.3389/fncel.2019.00159 31118887 PMC6504789

[B17] Feurer M. Klein A. Eggensperger K. Springenberg J. Blum M. Hutter F. (2015). Efficient and robust automated machine learning. Adv. neural Inf. Process. Syst. 28.

[B18] Gaita F. Giustetto C. Bianchi F. Schimpf R. Haissaguerre M. Calò L. (2004). Short QT syndrome: pharmacological treatment. J. Am. Coll. Cardiol. 43 (8), 1494–1499. 10.1016/j.jacc.2004.02.034 15093889

[B19] Garg P. Garg V. Shrestha R. Sanguinetti M. C. Kamp T. J. Wu J. C. (2018). Human induced pluripotent stem cell-derived cardiomyocytes as models for cardiac channelopathies: a primer for non-electrophysiologists. Circulation Res. 123 (2), 224–243. 10.1161/CIRCRESAHA.118.311209 29976690 PMC6136439

[B20] Georgiadis V. Stephanou A. Townsend P. A. Jackson T. R. (2015). MultiElec: a MATLAB based application for MEA data analysis. PLOS ONE 10 (6), e0129389. 10.1371/JOURNAL.PONE.0129389 26076010 PMC4468069

[B21] Hayes H. B. Nicolini A. M. Arrowood C. A. Chvatal S. A. Wolfson D. W. Cho H. C. (2019). Novel method for action potential measurements from intact cardiac monolayers with multiwell microelectrode array technology. Sci. Rep. 9 (1), 11893–11913. 10.1038/s41598-019-48174-5 31417144 PMC6695445

[B22] Iachetta G. Colistra N. Melle G. Deleye L. Tantussi F. De Angelis F. (2021). Improving reliability and reducing costs of cardiotoxicity assessments using laser-induced cell poration on microelectrode arrays. Toxicol. Appl. Pharmacol. 418, 115480. 10.1016/J.TAAP.2021.115480 33689843

[B23] Iachetta G. Melle G. Colistra N. Tantussi F. De Angelis F. Dipalo M. (2023). Long-term *in vitro* recording of cardiac action potentials on microelectrode arrays for chronic cardiotoxicity assessment. Archives Toxicol. 97 (2), 509–522. 10.1007/s00204-022-03422-y PMC9859891 36607357

[B24] Jahed Z. Yang Y. Tsai C. T. Foster E. P. McGuire A. F. Yang H. (2022). Nanocrown electrodes for parallel and robust intracellular recording of cardiomyocytes. Nat. Commun. 13 (1), 2253–2314. 2022/4//2022. 10.1038/s41467-022-29726-2 35474069 PMC9042818

[B25] Jans D. Callewaert G. Krylychkina O. Hoffman L. Gullo F. Prodanov D. (2017). Action potential-based MEA platform for *in vitro* screening of drug-induced cardiotoxicity using human iPSCs and rat neonatal myocytes. J. Pharmacol. Toxicol. Methods 87, 48–52. 10.1016/j.vascn.2017.05.003 28549786

[B26] Lee J. Gänswein T. Ulusan H. Emmenegger V. Saguner A. M. Duru F. (2022). Repeated and on-demand intracellular recordings of cardiomyocytes derived from human-induced pluripotent stem cells. ACS Sensors 7 (10), 3181–3191. 10.1021/acssensors.2c01678 36166837 PMC7613763

[B27] Li W. Stauske M. Luo X. Wagner S. Vollrath M. Mehnert C. S. (2020). Disease phenotypes and mechanisms of iPSC-derived cardiomyocytes from brugada syndrome patients with a loss-of-function SCN5A mutation. Front. Cell. Dev. Biol. 8, 592893–601181. 10.3389/fcell.2020.592893 33195263 PMC7642519

[B28] Lin Z. C. McGuire A. F. Burridge P. W. Matsa E. Lou H. Y. Wu J. C. (2017). Accurate nanoelectrode recording of human pluripotent stem cell-derived cardiomyocytes for assaying drugs and modeling disease. Microsystems Nanoeng. 3 (1), 16080–16087. 10.1038/micronano.2016.80 PMC6444980 31057850

[B29] Millard D. Dang Q. Shi H. Zhang X. Strock C. Kraushaar U. (2018). Cross-site reliability of human induced pluripotent stem cell-derived cardiomyocyte based safety assays using microelectrode arrays: results from a blinded CiPA pilot study. Toxicol. Sci. official J. Soc. Toxicol. 164 (2), 550–562. 10.1093/toxsci/kfy110 PMC6061700 29718449

[B30] Mulder P. de Korte T. Dragicevic E. Kraushaar U. Printemps R. Vlaming M. L. H. (2018). Predicting cardiac safety using human induced pluripotent stem cell-derived cardiomyocytes combined with multi-electrode array (MEA) technology: a conference report. J. Pharmacol. Toxicol. Methods 91, 36–42. 10.1016/J.VASCN.2018.01.003 29355722

[B31] Müller J. Ballini M. Livi P. Chen Y. Radivojevic M. Shadmani A. (2015). High-resolution CMOS MEA platform to study neurons at subcellular, cellular, and network levels. Lab a Chip 15 (13), 2767–2780. 10.1039/C5LC00133A PMC5421573 25973786

[B32] Multiple Cause (2024). Multiple cause of death 2018-2021 and provisional data 2022-2023 on CDC WONDER database. Centers Dis. Control Prev. Natl. Cent. Health Statistics. Natl. Vital Statistics Syst. Provisional Mortal. CDC WONDER Online Database. Available at: http://wonder.cdc.gov/mcd-icd10-provisional.html (Accessed July 28, 2023).

[B33] Pai V. B. Nahata M. C. (2000). Cardiotoxicity of chemotherapeutic agents: incidence, treatment and prevention. Drug Saf. 22, 263–302. 10.2165/00002018-200022040-00002 10789823

[B34] Pedregosa F. (2011). Scikit-learn: machine learning in Python. J. Mach. Learn. Res. 12 (85), 2825–2830.

[B35] Petch J. Di S. Nelson W. (2022). Opening the black box: the promise and limitations of explainable machine learning in cardiology. Can. J. Cardiol. 38 (2), 204–213. 10.1016/j.cjca.2021.09.004 34534619

[B36] Pradhapan P. Kuusela J. Viik J. Aalto-Setälä K. Hyttinen J. (2013). Cardiomyocyte MEA data analysis (CardioMDA) - a novel field potential data analysis software for pluripotent stem cell derived cardiomyocytes. PLoS ONE 8 (9), e73637. 10.1371/journal.pone.0073637 24069215 PMC3777951

[B37] Scheel O. Frech S. Amuzescu B. Eisfeld J. Lin K. H. Knott T. (2014). Action potential characterization of human induced pluripotent stem cell-derived cardiomyocytes using automated patch-clamp technology. Assay Drug Dev. Technol. 12 (8), 457–469. 10.1089/adt.2014.601 25353059

[B38] Shinnawi R. Shaheen N. Huber I. Shiti A. Arbel G. Gepstein A. (2019). Modeling reentry in the short QT syndrome with human-induced pluripotent stem cell–derived cardiac cell sheets. J. Am. Coll. Cardiol. 73 (18), 2310–2324. 10.1016/j.jacc.2019.02.055 31072576

[B39] Singal P. K. Iliskovic N. (1998). Doxorubicin-induced cardiomyopathy. N. Engl. J. Med. 339 (13), 900–905. 10.1056/NEJM199809243391307 9744975

[B40] Spira M. E. Hai A. (2013). Multi-electrode array technologies for neuroscience and cardiology. Nat. Nanotechnol. 8, 83–94. 10.1038/nnano.2012.265 23380931

[B41] Suzuki I. Matsuda N. Han X. Noji S. Shibata M. Nagafuku N. (2023). Large-area field potential imaging having single neuron resolution using 236 880 electrodes CMOS-MEA technology. Adv. Sci. 10 (20), e2207732. 10.1002/advs.202207732 PMC10369302 37088859

[B42] Wolpert C. Schimpf R. Giustetto C. Antzelevitch C. Cordeiro J. Dumaine R. (2005). Further insights into the effect of quinidine in short QT syndrome caused by a mutation in HERG. J. Cardiovasc. Electrophysiol. 16 (1), 54–58. 10.1046/j.1540-8167.2005.04470.x 15673388 PMC1474841

[B43] Yamamoto W. Asakura K. Ando H. Taniguchi T. Ojima A. Uda T. (2016). Electrophysiological characteristics of human iPSC-derived cardiomyocytes for the assessment of drug-induced proarrhythmic potential. PLOS ONE 11 (12), e0167348. 10.1371/journal.pone.0167348 27923051 PMC5140066

[B44] Yuan X. Schröter M. Obien M. E. J. Fiscella M. Gong W. Kikuchi T. (2020). Versatile live-cell activity analysis platform for characterization of neuronal dynamics at single-cell and network level. Nat. Commun. 11 (1), 4854. 10.1038/s41467-020-18620-4 32978383 PMC7519655

[B45] Zwi L. Caspi O. Arbel G. Huber I. Gepstein A. Park I. H. (2009). Cardiomyocyte differentiation of human induced pluripotent stem cells. Circulation 120 (15), 1513–1523. 10.1161/CIRCULATIONAHA.109.868885 19786631

