# Supplementary material for: CardioMEA: comprehensive data analysis platform for studying cardiac diseases and drug responses
Source: Front Physiol. 2024 Oct 30;15:1472126. doi: 10.3389/fphys.2024.1472126 (PMC11557525; doi:10.3389/fphys.2024.1472126)
Supplement: Supplementary file 1 [file Table1.docx]

**Supplementary Information**


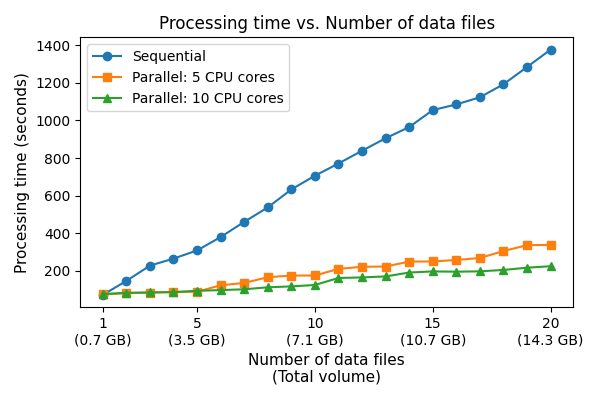


Figure S 1. The processing time massively increases as the number of data files grows. However, the increase is significantly reduced if parallel processing is employed using multiple CPUs.


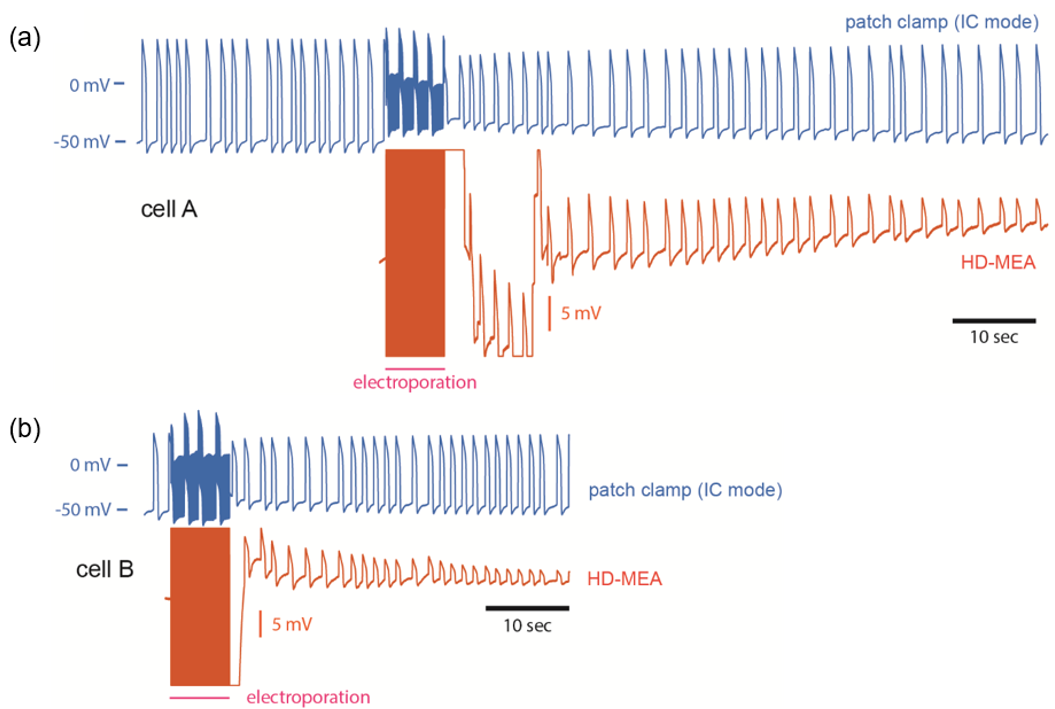


Figure S 2. Intracellular recordings of (a) cell A and (b) cell B by using whole-cell patch clamp in the current-clamp mode (blue) and intracellular-like recordings after HD-MEA upon electroporation (red). Both signal traces (patch and MEA) were simultaneously obtained from the same cell as can be seen from the decrease of the patch signal amplitude upon HD-MEA poration. Reprinted with permission from ACS Sensors 2022, 7, 10, 3181–3191. Copyright 2022 American Chemical Society.


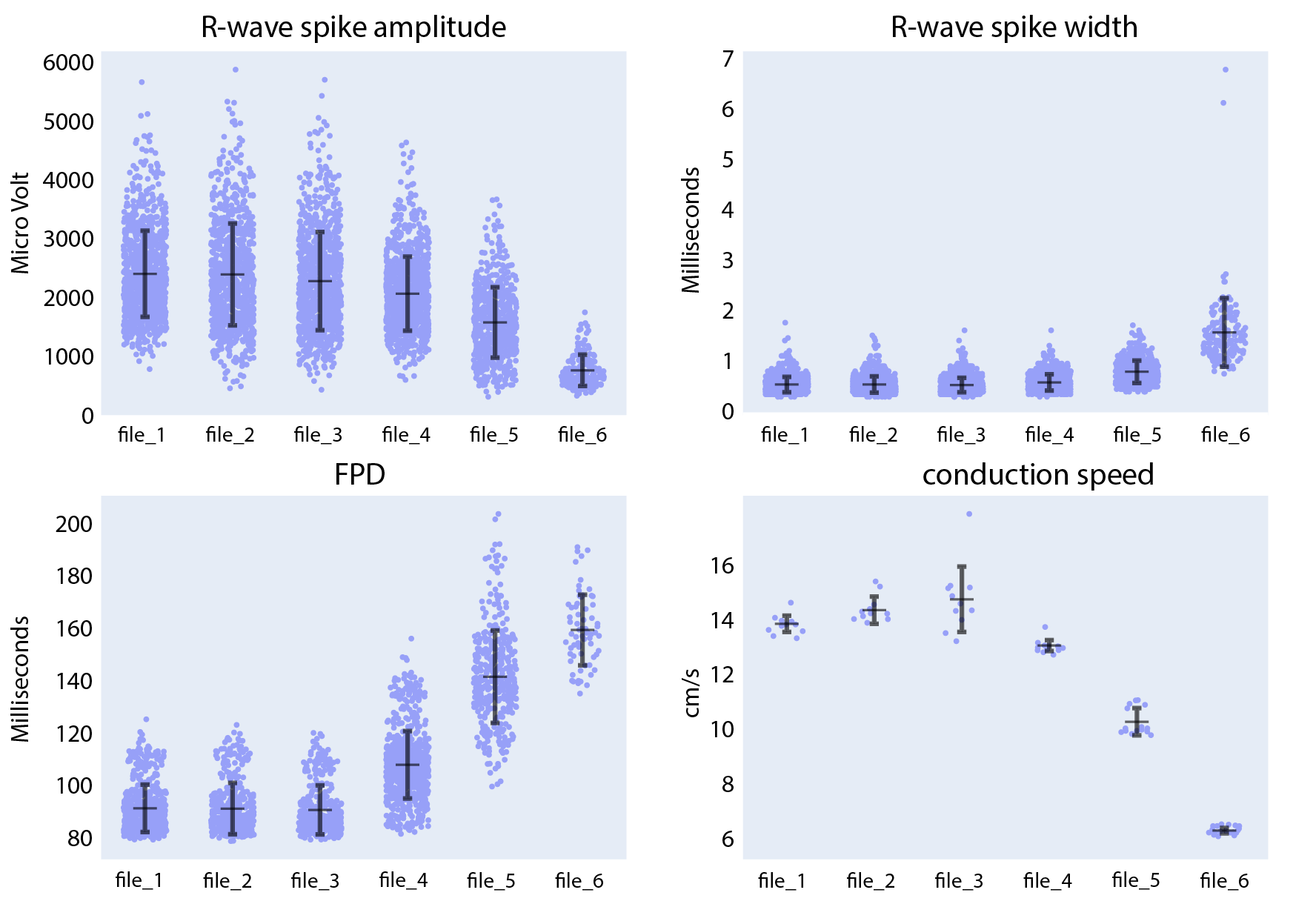


Figure S 3. Compound analysis of signals of CMs derived from SQT5-line iPSCs using the Extracellular Analysis panel. Following four initial baseline measurements (file_1 to file_3), the concentration of disopyramide was sequentially increased in the following sequence: 3 µM (file_4), 13 µM (file_5), 43 µM (file_6). Each data point in the provided figures corresponds to a value obtained from a single recording electrode. The horizontal and vertical bars denote the mean values and standard deviations (mean ± standard deviation), respectively.


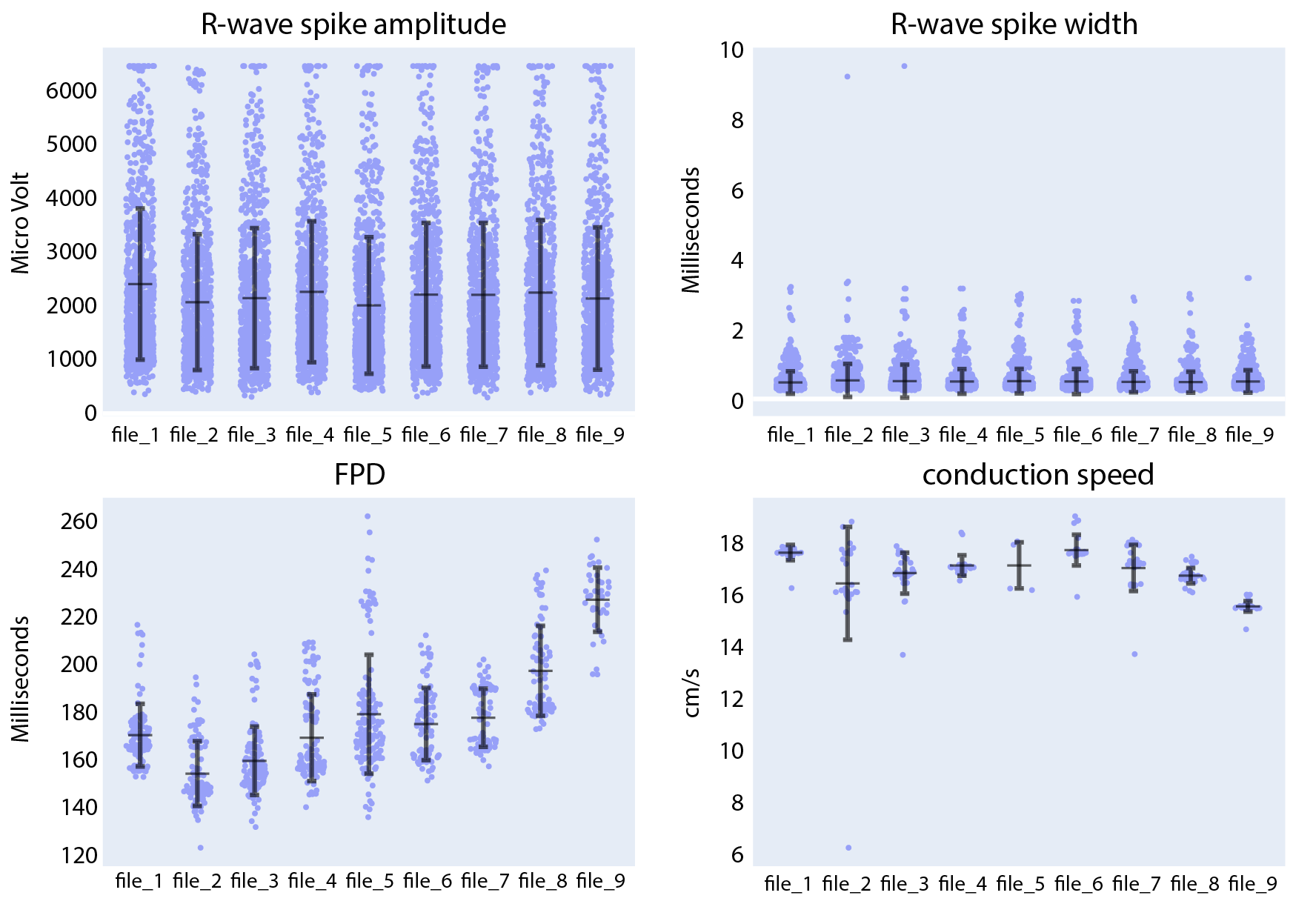


Figure S 4. Compound analysis of signals of CMs derived from SQT5-line iPSCs using the Extracellular Analysis panel. Following four initial baseline measurements (file_1 to file_4), the concentration of sotalol was sequentially increased in the following sequence: 3 µM (file_5), 10 µM (file_6), 30 µM (file_7), 100 µM (file_8), 300 µM (file_9). Each data point in the provided figures corresponds to a value obtained from a single recording electrode. The horizontal and vertical bars denote the mean values and standard deviations (mean ± standard deviation), respectively. In the R-wave spike amplitude plot, the clipped data points represent 1.6% of the data in the first baseline measurement.


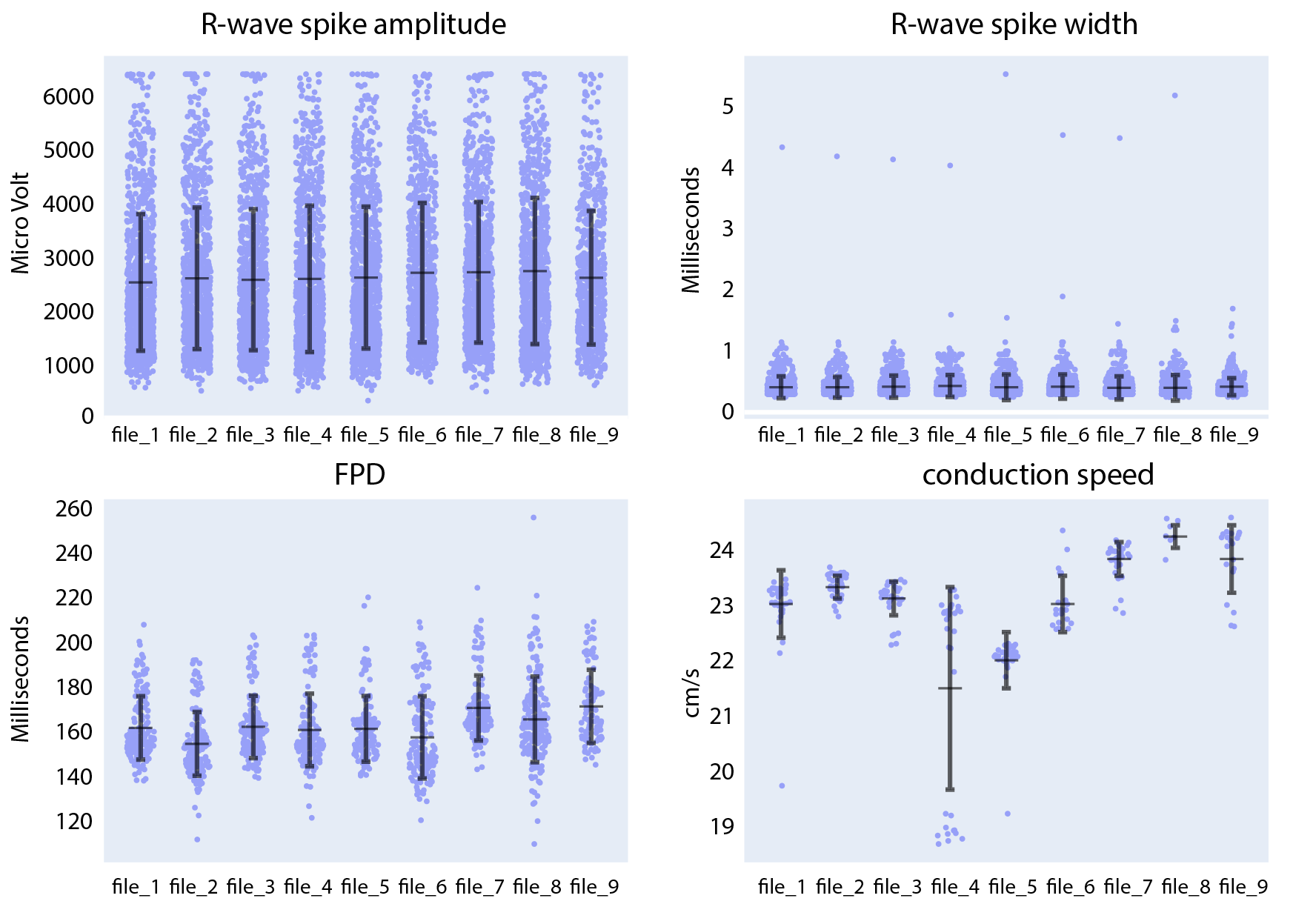


Figure S 5. Compound analysis of signals of CMs derived from SQT5-line iPSCs using the Extracellular Analysis panel. Following four initial baseline measurements (file_1 to file_4), the concentration of flecainide was sequentially increased in the following sequence: 0.03 µM (file_5), 0.1 µM (file_6), 0.3 µM (file_7), 1 µM (file_8), 3 µM (file_9). Each data point in the provided figures corresponds to a value obtained from a single recording electrode. The horizontal and vertical bars denote the mean values and standard deviations (mean ± standard deviation), respectively. In the R-wave spike amplitude plot, the clipped data points represent 0.4% of the data in the first baseline measurement.


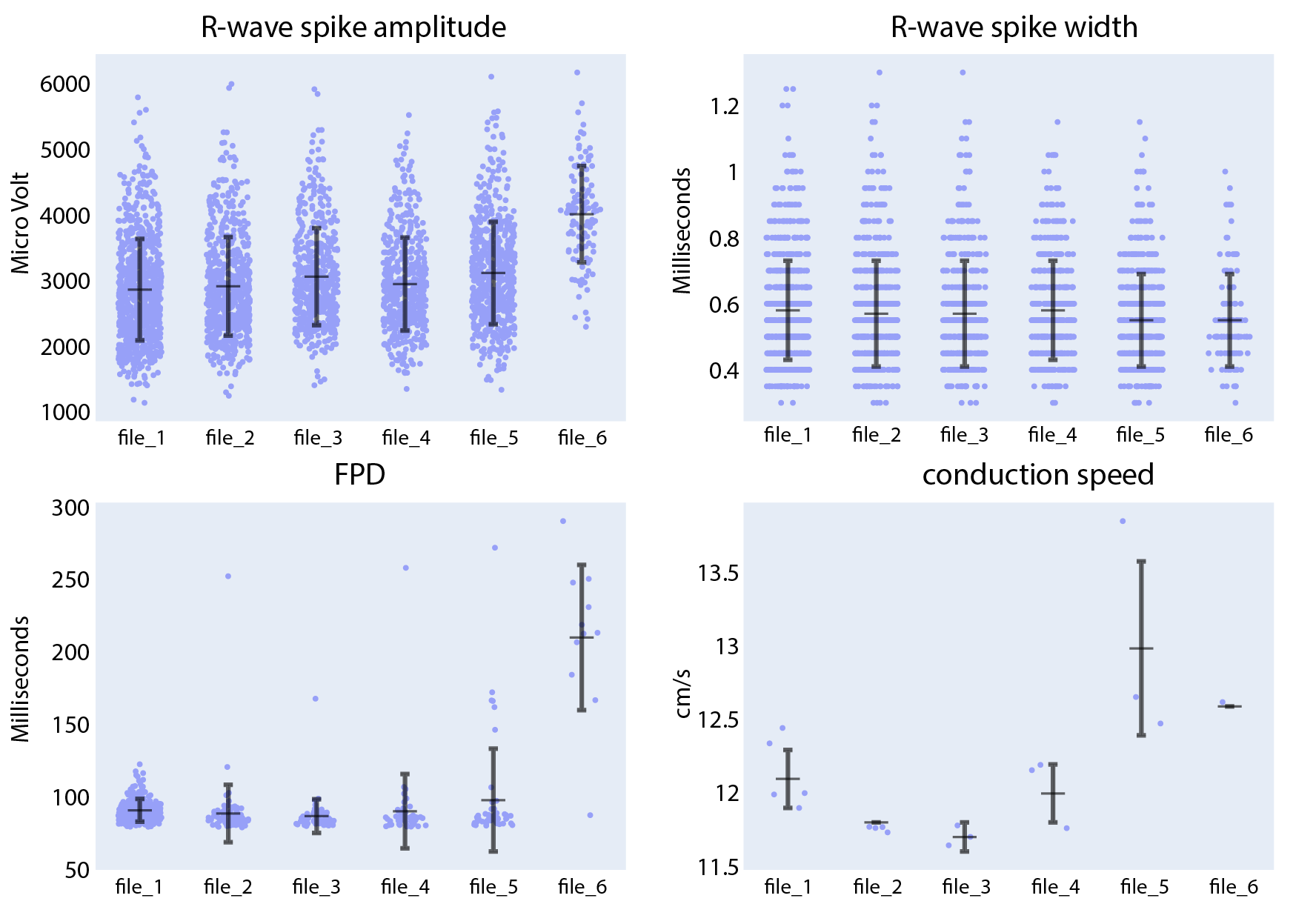


Figure S 6. Compound analysis of signals of CMs derived from SQT5-line iPSCs using the Extracellular Analysis panel. Following four initial baseline measurements (file_1 to file_3), the concentration of ivabradine was sequentially increased in the following sequence: 0.3 µM (file_4), 1 µM (file_5), 3 µM (file_6). Each data point in the provided figures corresponds to a value obtained from a single recording electrode. The horizontal and vertical bars denote the mean values and standard deviations (mean ± standard deviation), respectively.


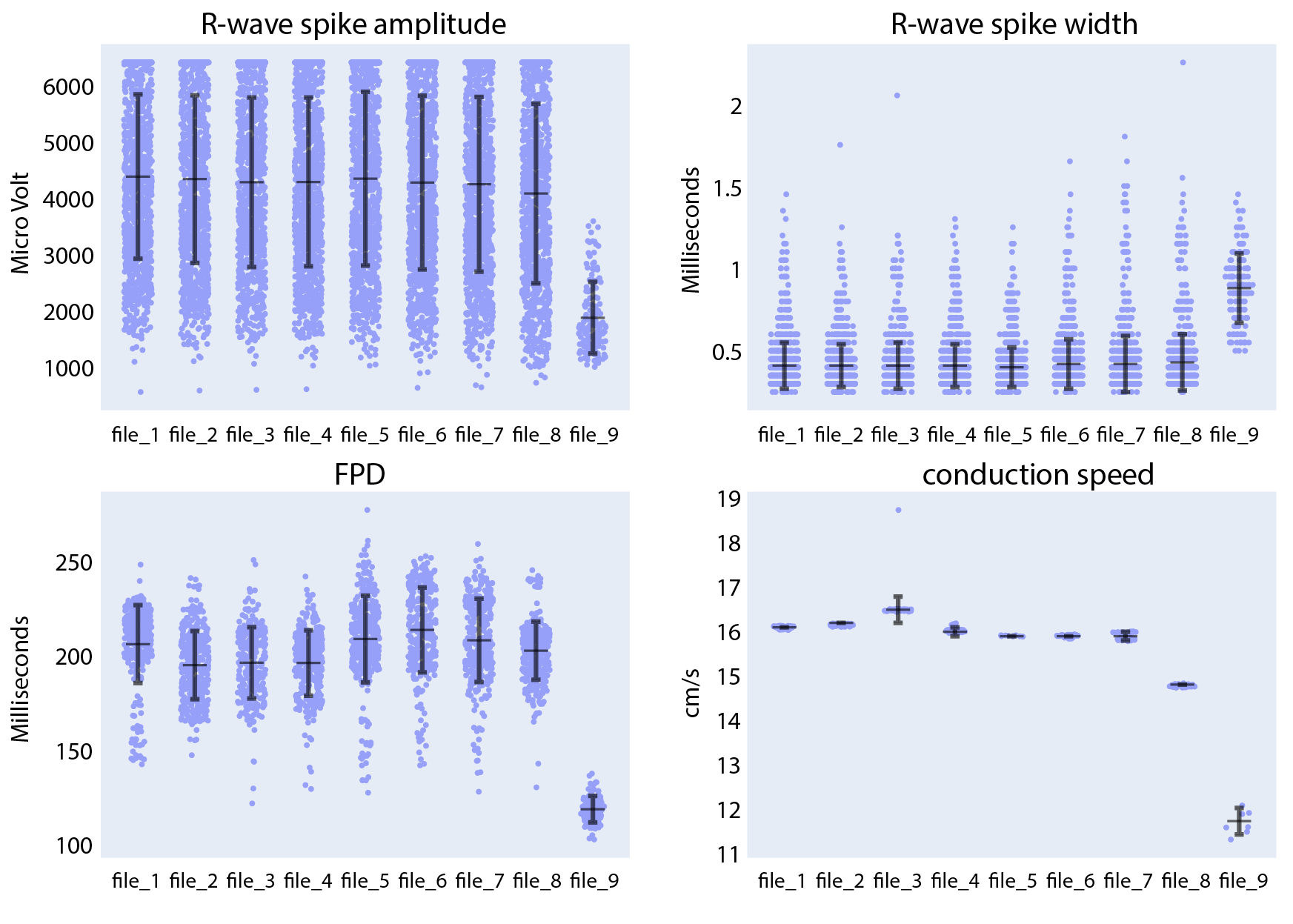


Figure S 7. Compound analysis of signals of CMs derived from SQT5-line iPSCs using the Extracellular Analysis panel. Following four initial baseline measurements (file_1 to file_4), the concentration of amiodarone was sequentially increased in the following sequence: 0.03 µM (file_5), 0.1 µM (file_6), 0.3 µM (file_7), 1 µM (file_8), 3 µM (file_9). Each data point in the provided figures corresponds to a value obtained from a single recording electrode. The horizontal and vertical bars denote the mean values and standard deviations (mean ± standard deviation), respectively. In the R-wave spike amplitude plot, the clipped data points represent 15.7% of the data in the first baseline measurement.


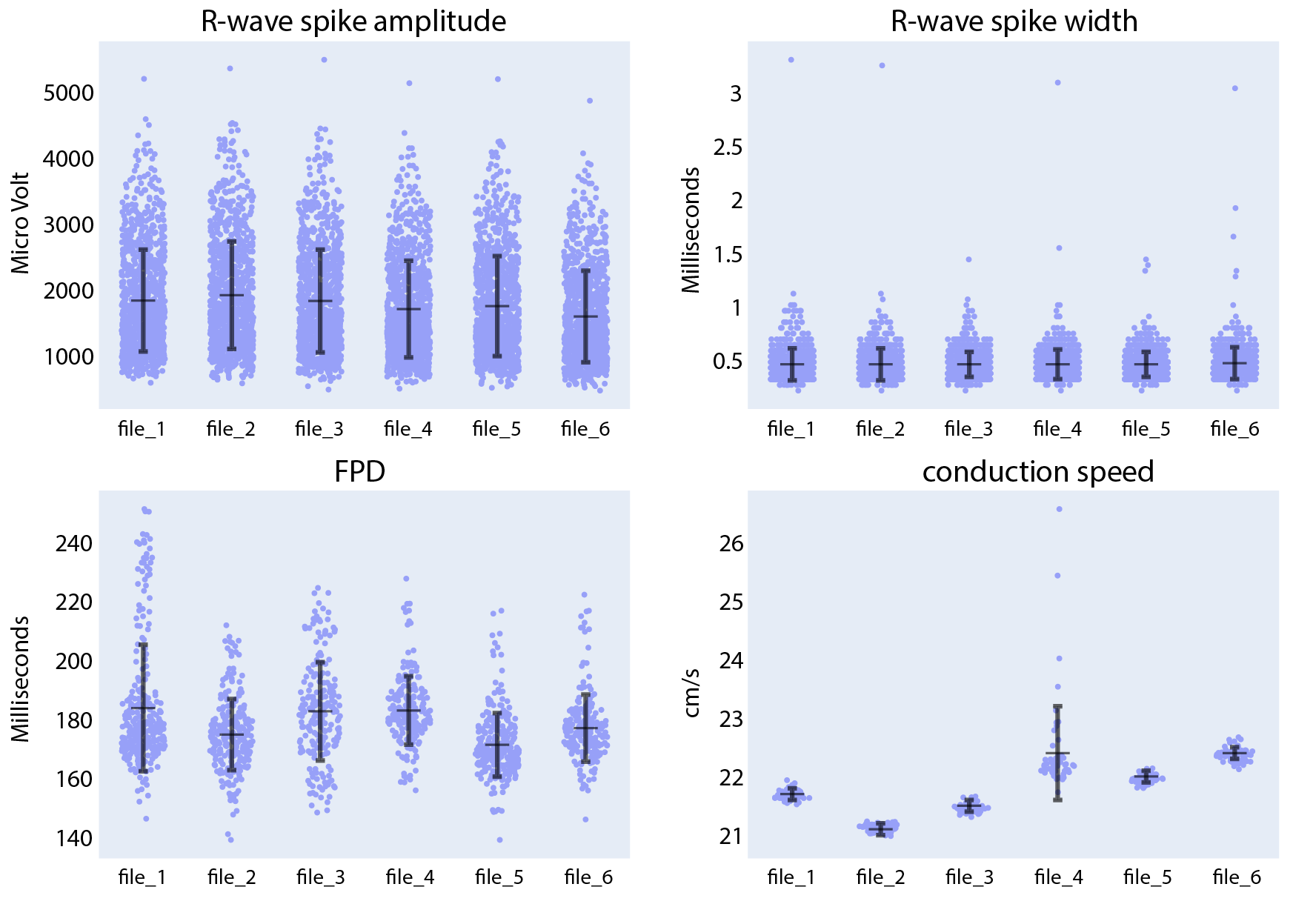


Figure S 8. Compound analysis of signals of CMs derived from SQT5-line iPSCs using the Extracellular Analysis panel. Following four initial baseline measurements (file_1 to file_3), the concentration of ranolazine was sequentially increased in the following sequence: 0.3 µM (file_4), 1 µM (file_5), 3 µM (file_6). Each data point in the provided figures corresponds to a value obtained from a single recording electrode. The horizontal and vertical bars denote the mean values and standard deviations (mean ± standard deviation), respectively.


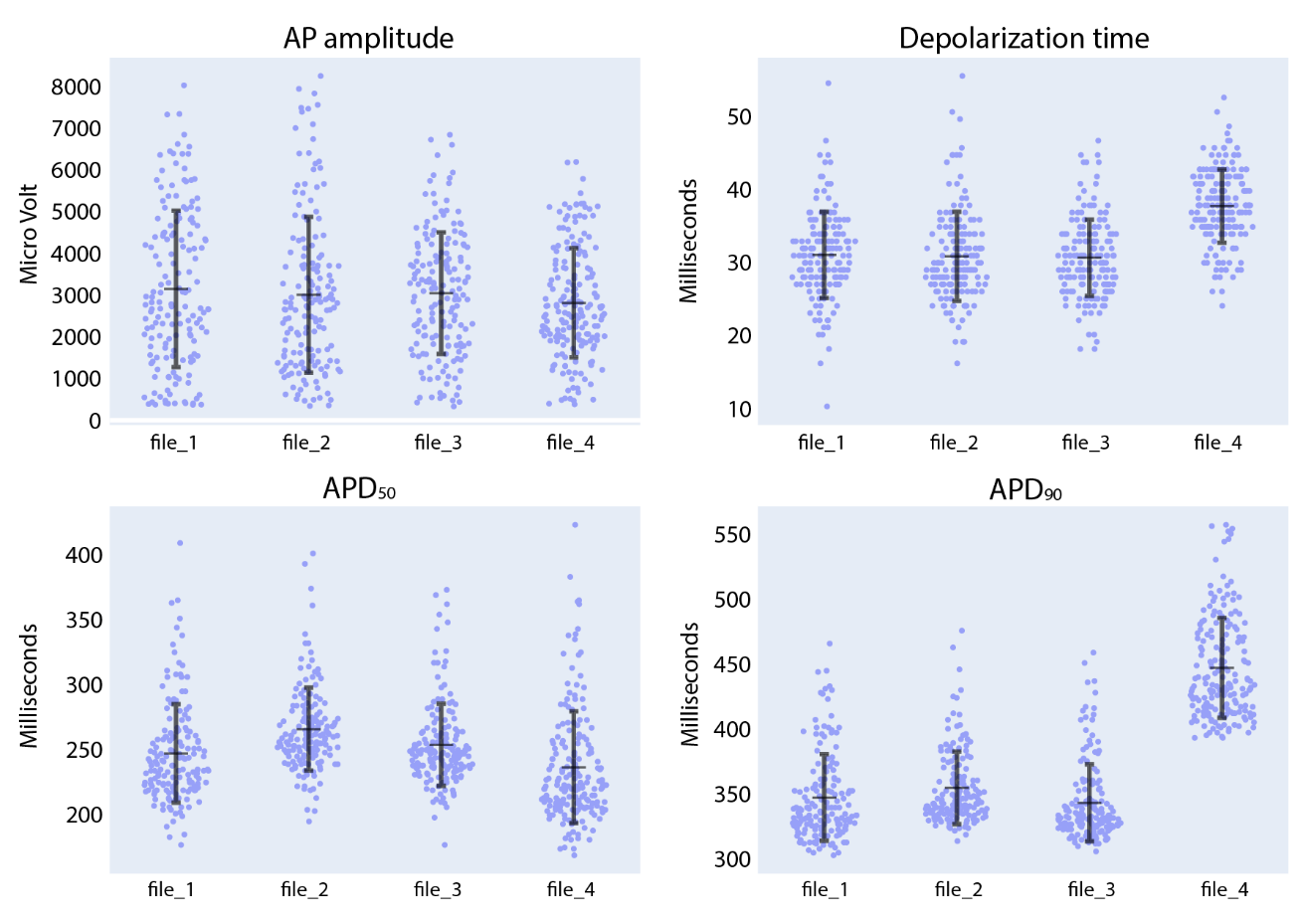


Figure S 9. Compound analysis of signals of iCell Cardiomyocytes using the Intracellular Analysis panel. Following three initial baseline measurements (file_1 to file_3), quinidine was added to the culture to reach a concentration of 1 µM (file_4). Each data point in the provided figures corresponds to a value obtained from a single recording electrode. The horizontal and vertical bars denote the mean value and standard deviation (mean ± standard deviation), respectively.


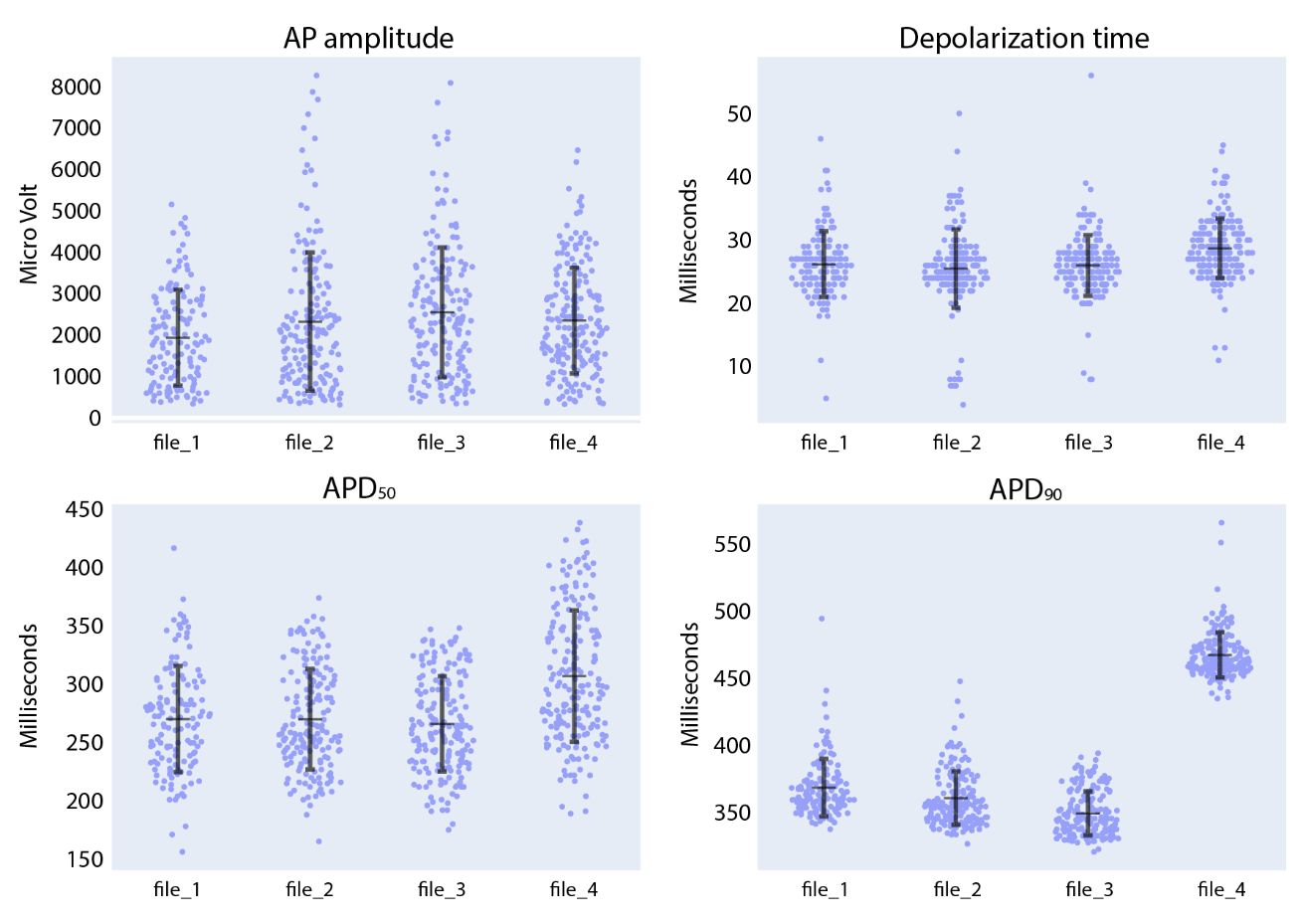


Figure S 10. Compound analysis of signals of iCell Cardiomyocytes using the Intracellular Analysis panel. Following three initial baseline measurements (file_1 to file_3), sotalol was added to the culture to reach a concentration of 30 µM (file_4). Each data point in the provided figures corresponds to a value obtained from a single recording electrode. The horizontal and vertical bars denote the mean value and standard deviation (mean ± standard deviation), respectively.


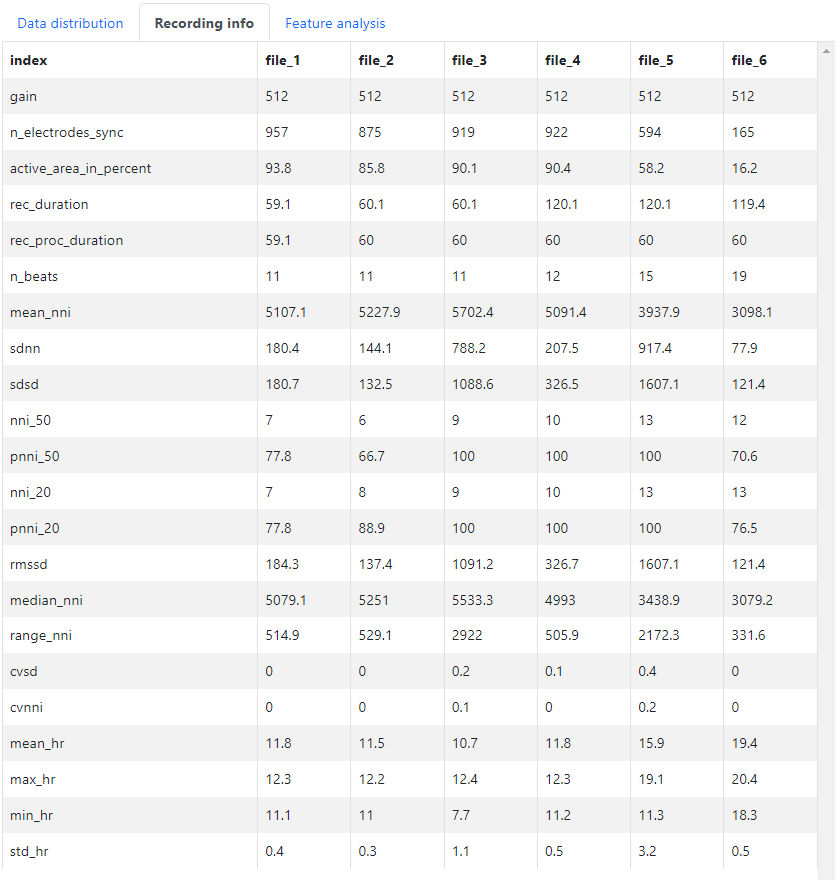


Figure S 11. Detailed information on each recording file can be found under the “Recording info” tab in the CardioMEA Dashboard. The image shows data generated during disopyramide administration to CMs derived from SQT5-line iPSCs. Following four initial baseline measurements (file_1 to file_3), the concentration of disopyramide was sequentially increased in the following sequence: 3 µM (file_4), 13 µM (file_5), 43 µM (file_6).

Supplementary Method 1. Voltage Electroporation Protocol

The protocol and the conditions under which intracellular-like signals were obtained are described below. This text is reprinted with permission from ACS Sensors 2022, 7, 10, 3181–3191. Copyright 2022 American Chemical Society.

All extracellular and intracellular measurements were performed in a humidified, 5% CO2 incubator at 37 °C. HD-MEA chips were mounted on MaxOne Recording Units (MaxWell Biosystems AG, Zurich, Switzerland) for data acquisition and control of electroporation.

Data acquisition and electroporations were performed using the MaxLive Software (version 19.2.27, MaxWell Biosystems AG, Zurich, Switzerland) in conjunction with a custom-written Python script for API controls. For electroporation, the selected electrodes were connected to on-chip stimulation/poration units. The core of each unit was a class-AB opamp, capable of driving loads as large as 10 nF, while maintaining a low static power consumption (for details, see [1]). For voltage stimulation, the circuit was configured as an inverting amplifier with low output impedance [1]. We applied rectangular biphasic voltage pulses of 1−2 V peak-to-peak amplitude (Vpp) and 50 μs to 1 ms phase duration at 1 ms interpulse intervals (IPIs). The poration duration was maximally 35 s. Although the applied voltage amplitude exceeded that of the water window in some instances, the frequency of the AC signal was large enough (333 Hz to 10 kHz range) to not produce electrolysis, and we did not observe any signs of gas evolution. After delivery of the respective voltage pulses, electrodes were disconnected from the stimulation units to perform the voltage recordings. Up to 990 electrodes could be simultaneously connected to stimulation units to perform voltage stimulation.

Supplementary Method 2. AUTO-SKLEARN Classification Algorithms

No single machine learning model consistently outperformed the others across all datasets, which necessitates a selection and optimization of the model for each specific data scenario. This process requires expert knowledge and can be time-consuming. Automated machine learning (AutoML) addresses these challenges by systematically evaluating various models and tuning hyperparameters, thereby identifying the most suitable model for a given dataset. AUTO-SKLEARN is an open-source, AutoML toolkit that uses the well-known Scikit-Learn library. Table S 1. shows a list of models used by AUTO-SKLEARN and descriptions of each model [2, 3]. AUTO-SKLEARN utilizes combined model selection and hyperparameter optimization using Bayesian optimization, recognizing that the performance of machine learning models may heavily depend on the tuning of hyperparameters. The default metric used by AUTO-SKLEARN for evaluating classifier models is accuracy, which can be replaced by other built-in metrics depending on the use-case [4].

Table S 1. Models used by AUTO-SKLEARN classification algorithms [2, 3]

| Model name | Description |
| --- | --- |
| AdaBoost | Ensemble method that combines weak classifiers to form a strong classifier. It focuses on misclassified instances in subsequent iterations to improve overall accuracy. |
| Decision tree | Splits the data into subsets recursively based on the most informative features. Each leaf node represents a class label. |
| Gradient Boosting Machine | Ensemble method where each successive tree addresses the mistakes of the previous ones, leading to improved performance. |
| Random forest | Ensemble method that boosts performance by using multiple decision trees built by randomly-chosen features. |
| Extremely randomized trees | Similar to random forests but introduces more randomness in the way splits are chosen. |
| Gaussian Naive Bayes | Probabilistic model with the assumption that features follow a Gaussian distribution and are independent given the class label. |
| Bernoulli naive Bayes | Unlike Gaussian Naive Bayes, this model assumes that the features are binary. The features are assumed to be independent. |
| Multinomial naive Bayes | This model is suited for data where the features represent counts. The features are assumed to be independent. |
| k-Nearest Neighbors | Predicts class based on the majority class of the k-nearest neighbors in the training set. |
| Linear Support Vector Machine | Support vector classifier with a linear kernel, that finds the hyperplane that best separates the classes in the feature space. |
| Kernel Support Vector Machine | Applies kernel functions to transform the feature space so that linear separation is possible. |
| Linear Discriminant Analysis | Projects the data onto a lower-dimensional space while maximizing the separation between the classes. It assumes that the classes share the same covariance matrix. |
| Quadratic Discriminant Analysis | Similar to Linear Discriminant Analysis, but it assumes that each class has its own covariance matrix. Suitable for datasets where classes are not linearly separable. |
| passive aggressive | Online learning method that updates the model only when a new data point is incorrectly classified. |
| Linear Classifier (SGD) | Linear model, i.e., logistic regression, support vector machine, etc., with stochastic gradient descent (SGD) learning to optimize the loss function. |
| Multi-Layer Perceptron | Artificial neural network made up of layers of neurons. Each neuron in a layer applies a weighted sum followed by a non-linear activation function to produce an output. |

**References**

[1] M. Ballini et al., "A 1024-Channel CMOS Microelectrode Array With 26,400 Electrodes for Recording and Stimulation of Electrogenic Cells In Vitro," IEEE Journal of Solid-State Circuits, vol. 49, no. 11, pp. 2705-2719, 2014/11// 2014, doi: 10.1109/JSSC.2014.2359219.

[2] M. Feurer, A. Klein, K. Eggensperger, J. Springenberg, M. Blum, and F. Hutter, "Efficient and robust automated machine learning," Advances in neural information processing systems, vol. 28, 2015.

[3] “Scikit-Learn User Guide: Supervised learning”, <https://scikit-learn.org/stable/supervised_learning.html> (accessed 2024-09-01).

[4] “AUTO-SKLEARN documentation: Build-in Metrics”, <https://automl.github.io/auto-sklearn/master/api.html#built-in-metrics> (accessed 2024-09-01).
